# Integrated Virtual Screening and Molecular Electrostatic Surface Potential Descriptors for Predicting BACE1 Inhibitor Interactions in Alzheimer’s Disease

**DOI:** 10.1021/acsomega.6c02969

**Published:** 2026-08-07

**Authors:** Yoanna Alvarez-Ginarte, Rafael López, José Manuel García de la Vega, Ignacio De Ema, Daniel Alpízar-Pedraza

**Affiliations:** † Universidad de la Habana, Facultad de Química, Havana 10400, Cuba; ‡ 16722 Universidad Autónoma de Madrid, Química Física Aplicada, Madrid, 28049 Madrid, Spain; § Center for Pharmaceuticals Research and Development (CIDEM), La Habana 10400, Cuba

## Abstract

Developing brain-penetrant BACE1 inhibitors remains a major challenge in Alzheimer’s disease. Unlike conventional studies focusing solely on binding affinity, this work presents an integrated computational pipeline that prioritizes both potency and pharmacokinetic developability from the outset. A robust quantitative structure–activity relationship (QSAR) model was built for 41 N-(pyridin-3-yl)­picolinamide derivatives using Molecular Electrostatic Surface Potential (MESP) descriptors. Principal component analysis identified three key electrostatic features – mean MESP, variance of negative MESP, and number of local MESP maxima/minima as drivers of BACE1 inhibitory activity. A key novelty is the early integration of Absorption, Distribution, Metabolism, Excretion and Toxicity (ADMET) filtering, using ADMETlab 3.0, to ensure blood-brain barrier (BBB) permeability and low P-glycoprotein efflux properties whose absence contributed to the failure of several clinical BACE1 inhibitors. Only compound N-[3-[(1S,5S,6S)-3-amino-1-(difluoromethyl)-5-(fluoromethyl)-2-thia-4-azabicyclo[4.1.0]­hept-3-en-5-yl]-4-fluorophenyl]-5-chloropyrazine-2-carboxamide (5) simultaneously satisfied the criteria for high predicted potency, favorable BBB penetration, low PgP efflux, and good synthetic accessibility. Molecular docking revealed that compound 5 binds strategically to the S1 and S3 substrate-recognition pockets through hydrogen bonds with THR280, GLN121, LYS155, as well as a halogen interaction with ARG283, while avoiding direct interaction with the catalytic dyad. This binding mode balances affinity and permeability, distinguishing compound 5 from clinically unsuccessful inhibitors. The practical implication is a rational framework that rapidly prioritizes candidates with high potency and favorable Central Nervous System (CNS) drug-like properties, thereby reducing the risk of late-stage attrition. All predictions are computational and require experimental validation (enzymatic, cellular, and in vivo) before any translational claims can be made. Thus, combining QSAR, ADMET, and molecular docking with an early emphasis on BBB penetration offers a time-efficient strategy for CNS drug discovery against Alzheimer’s disease.

## Graphical abstract

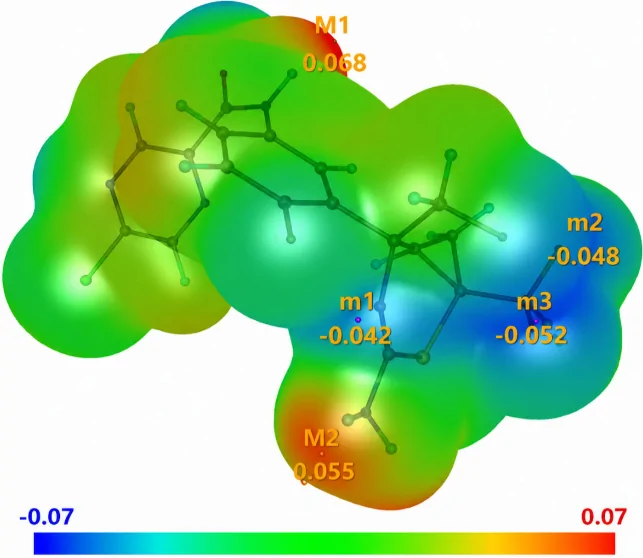

## 1 Introduction

Alzheimer’s disease is an increasingly prevalent disorder as a consequence of population aging, particularly in developed countries, and it has a significant social impact. For this reason, considerable efforts are currently being devoted to investigating its causes and potential treatments to alleviate its effects, combining experimental, clinical, and theoretical approaches.^1^

From a biochemical perspective, Alzheimer’s disease can be understood as a pathological cascade triggered by abnormal protein processing, in which the proteolysis of the amyloid precursor protein (APP) plays a fundamental initiating role. This membrane protein can be degraded through two competing enzymatic pathways. The nonamyloidogenic pathway, mediated by alpha-secretase, produces harmless fragments and predominates in the healthy brain.^2,3^ However, in the disease state, the amyloidogenic pathway is enhanced, and its rate-limiting and decisive step is catalyzed by the enzyme beta-secretase 1 (BACE1).^4^ This enzyme performs the primary cleavage of APP, generating a substrate that is subsequently processed by gamma-secretase to release the beta-amyloid peptide (Aβ), particularly the Aβ42 isoform. This peptide aggregates to form toxic oligomers and senile plaques, which disrupt neuronal communication and create a pro-inflammatory environment.^5^

Nevertheless, neuronal destruction and clinical symptoms are directly linked to the tau protein.^6^ Amyloid toxicity promotes tau hyperphosphorylation, causing its aggregation into neurofibrillary tangles within neurons, thereby disrupting the intracellular transport system and ultimately leading to cell death. The spread of these tangles marks the progression of dementia. Thus, although tau pathology acts as the ultimate executor of neurodegeneration, the trigger of the entire cascade lies in the critical involvement of BACE1. Its activity regulates the entry point into the amyloidogenic pathway, determining the production of the initial Aβ species that initiate the pathological process. Therefore, BACE1 represents a key therapeutic target, as inhibiting its action could prevent the first causal step in amyloid generation and consequently halt or mitigate the sequence of events leading to neurodegeneration and the cognitive deficits characteristic of Alzheimer’s disease. Owing to its crucial role, considerable research efforts have been devoted to developing BACE1 inhibitors as a therapeutic strategy.^4,7^

These inhibitors are designed to block BACE1 activity in order to reduce β-amyloid production and, in theory, slow or halt disease progression.^8–10^ However, clinical trials have encountered significant challenges, including adverse effects and limited efficacy in the advanced stages of the disease.^5^

Developing a new drug can take 9 to 13 years.^11^ Consequently computational methods have been increasingly adopted in recent decades to shorten this timeline. These methods have proven highly valuable for investigating biologically relevant phenomena and for the rational design of bioactive compounds.^12^ In particular, the integration of artificial intelligence (AI) and systems pharmacology approaches has transformed central nervous system (CNS) drug discovery. AI-assisted workflows now enable rapid prediction of blood–brain barrier permeability (BBB), off-target toxicity, and polypharmacology, while systems pharmacology models provide a holistic view of disease networks rather than focusing on single targets.^13,14^ Integrated molecular networking strategies have been successfully applied to identify novel bioactive compounds and to repurpose existing drugs for Alzheimer’s disease.^15,16^ These methodological innovations are particularly relevant for BACE1 inhibitor design, as they address the multifactorial nature of Alzheimer’s pathology and the need for compounds with balanced potency and CNS penetration.^17^

Among these computational strategies, virtual screening combined with machine learning (ML) and deep learning has emerged as a powerful tool for prioritizing lead compounds.^18,19^ Recent advances include a novel AI-driven drug-repurposing method (DeepDrug) for identifying promising combinations of approved drugs within the applicability domain (AD)^20^ as well as an integrated generative framework that combines ligand-based reinforcement learning with structure-based genetic algorithms to explore novel chemical space for potent and drug-like BACE1 inhibitors.^21^

In drug-receptor interactions, electrostatic attractions and repulsions play an essential role. The distribution of molecular electrostatic surface potential (MESP) within a drug molecule determines how it interacts with the active site of a biological receptor. Therefore, strong ligand–receptor binding is favored by good complementarity between the MESP distribution of the ligand and that of the receptor.^22^ Descriptors based on MESP capture information about the distribution of charge and reactivity, making them valuable for correlating electronic characteristics with biological effects and physicochemical properties. They are widely used in quantitative structure–activity relationship (QSAR) models to predict the pharmacological activity of molecules.^23^

The quantum chemical optimization of large polyatomic systems, such as weakly interacting protein–ligand complexes, is increasingly computationally demanding due to the high computational scaling of the methods involved, slow convergence of iterative procedures, and the large number of closely spaced local minima on the potential energy surface. In particular, the optimization of protein–ligand complexes requires bridging the accuracy of quantum mechanics (QM) with the scale of biological systems.This challenge is addressed through techniques such as fragmentation methods (fragment molecular orbital and molecular fractionation with conjugate caps) as well as hybrid approaches combining QM with molecular mechanics (QM/MM methods) and ML potentials trained on QM data. These strategies enable the treatment of systems containing large numbers of atoms and allow accurate prediction of binding energies by efficiently capturing complex electronic interactions-such as electrostatics and dispersion effects-that are often neglected by classical methods but are crucial for drug design.^24,25^

Unlike conventional high-throughput molecular docking, which relies on empirical scoring functions and often neglects global electronic distributions, our MESP-based QSAR approach provides a direct physicochemical interpretation of the electrostatic determinants of the activity. While docking is indispensable for visualizing specific ligand–receptor interactions, it does not quantitatively capture how the overall MESP distribution correlates with inhibitory potency across a series of analogues. Molecular dynamics simulations, although powerful for exploring conformational ensembles and binding stability, remain computationally expensive for rapid screening. Therefore, we integrate the speed and interpretability of MESP-derived descriptors with docking validation, providing a balanced strategy that is both mechanistically informative and computationally efficient. This hybrid approach allows us to identify key electronic features through QSAR analysis and subsequently use docking to rationalize the binding modes of the most promising candidates. The combined use of QSAR and molecular docking has been successfully applied to optimize inhibitors of various therapeutic targets, including kinases, demonstrating the utility of this integrated strategy for drug design.^26^

In the present work, we focus on predicting molecular interactions in BACE1 inhibitors for the treatment of Alzheimer’s disease by combining ligand-based and structure-based virtual screening methods with the evaluation of drug-like properties, together with the calculation of MESP-derived molecular descriptors.

The main objectives of this study are (1) to build predictive QSAR models based on principal component regression (PCR) for BACE1 inhibitors in order to determine which MESP-derived molecular descriptors exert the greatest influence on their experimental activity; (2) to estimate *in silico* pharmacokinetic properties, including BBB permeability and drug-likeness; (3) to perform docking simulations to achieve a comprehensive interpretation of the molecular interactions between the inhibitors and the target enzyme; and (4) to integrate the results from QSAR, docking, and absorption, distribution, metabolism, excretion, and toxicity (ADMET) analyses in order to explain the influence of MESP-derived descriptors on the interaction of these inhibitors with BACE1.

## 2 Computational Methods

A data set of 41 compounds sharing a common N-(pyridin-3-yl)­picolinamide substructure and exhibiting experimental inhibitory activity (IC_50_) against BACE1 was compiled from the literature (Table 1).^27,28^

**Table 1:**
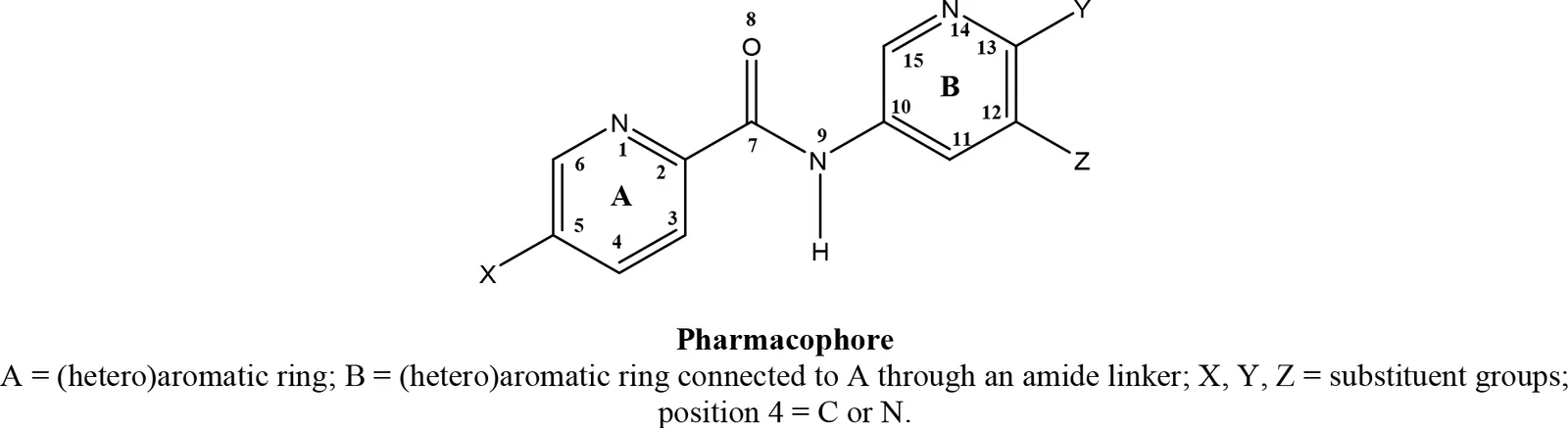

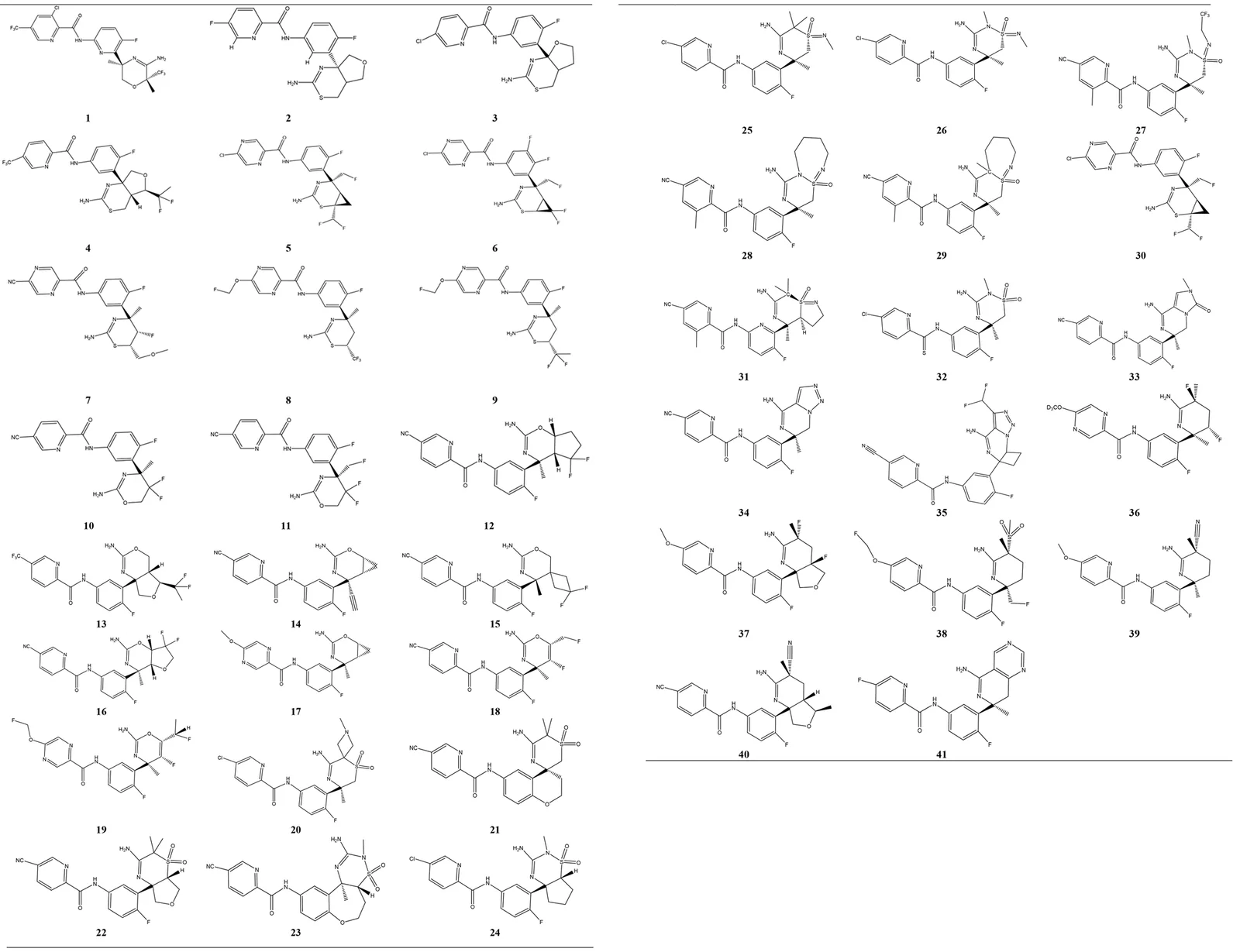
Two-Dimensional Structures of the 41 N-(Pyridin-3-yl)­picolinamide Derivatives Used as the Data Set in This Study

The negative decimal logarithm of IC_50_ (pIC_50_) was used as the dependent variable for QSAR modeling.^29^ Table S1 presents the experimental IC_50_ and pIC_50_ values. Figure 1 shows that the experimental pIC_50_ values range from 7.09 (compound 17) to 9.70 (compound 29). Molecular structures were generated using MOLPRO^30^ and optimized at the B3LYP/aug-σDZ level.^31–33^

**Figure 1:**
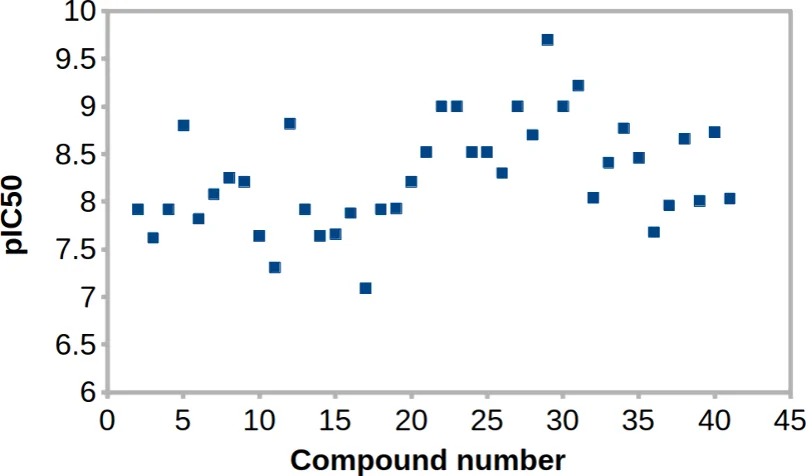
Distribution of experimental pIC_50_ values for the 41 N-(pyridin-3-yl)­picolinamide derivatives. Each point represents a single compound (numbered as in Table 1). The pIC_50_ values range from 7.09 (compound 17) to 9.70 (compound 29).

The exclusive use of isolated, gas-phase-optimized conformations for generating molecular descriptors in this 2D QSAR study, which employs global molecular properties, is a common and well-established practice.^34^

The bioactive conformation of most compounds in a data set is typically unknown, making geometry optimization in a receptor-free environment a standard approach for the systematic and unbiased generation of three-dimensional descriptors for correlation with biological activity. This strategy balances the predictive power and computational efficiency and has been successfully employed in numerous prior QSAR studies of CNS drug candidates.^35^

Using the DAMQT program,^36^ 19 molecular descriptors based on the MESP were calculated for each optimized structure. MESP can be efficiently computed on a molecular surface defined as an electron-density isosurface, typically at 0.001 au.^36,37^ These descriptors included: total area (*A*), volume (*Vol*), MESP maximum (*V*_*M*_), MESP minimum (*V*_*m*_), the number of disjoint regions containing maxima and minima (*Nmax*, *Nmin)*, approximate molecular dimensions (*δx, δy, δz)*, positive and negative areas (*A* ^+^, *A* ^–^), mean MESP (*V̅*), positive and negative mean MESP ( $\overset{®}{V^{+}\;}$ , $\overset{®}{V^{-}\;}$ ), MESP variance (σ^2^), positive and negative MESP variances ((σ ^+^)^2^ and (σ ^–^)^2^), MESP deviation (Π), and the ν parameter. Table S1 shows the full list of descriptors and their values.

Prior to principal component analysis (PCA), the descriptor matrix was mean-centered and scaled to unit variance (autoscaled) to ensure that all descriptors contributed equally to the analysis, regardless of their original magnitudes. To mitigate the risk of chance correlations and overfitting, the internal predictive ability of the model was evaluated through leave-one-out cross-validation (q^2^ > 0.5), and its external predictive power was assessed using an independent test set (R > 0.9). In addition, a Y-randomization test was performed by randomly shuffling the pIC_50_ values while keeping the descriptor matrix unchanged, thereby statistically assessing the possibility of chance correlations. To further define the chemical space within which the model is reliable, the applicability domain was characterized by using the leverage (hat) matrix, complementing standard residual-based outlier detection. All QSAR modeling procedures were in accordance with the Organization for Economic Co-operation and Development (OECD) principles for model validation, including a defined end point, an unambiguous algorithm, a defined applicability domain, appropriate measures of goodness-of-fit, robustness, and predictivity (R^2^, q^2^, R-test), and, whenever possible, a mechanistic interpretation.^38^

PCA was subsequently applied to the normalized descriptor matrix to reduce dimensionality and eliminate multicollinearity; three principal components (PCs) explaining most of the variance were retained. Descriptors exhibiting high loadings on the first PCs were considered relevant for biological activity.^39^ PCA was performed using BuildQSAR.^40^

Hierarchical cluster analysis (complete linkage, squared Euclidean distance) was performed on the descriptor matrix using STATISTICA 8 ^41^ to assess the structural diversity. Subsequently, nonhierarchical k-means clustering^42^ was employed to randomly divide the data set into a training set (80% of the compounds) and an external test set (20%), ensuring that the latter was not used during model training.^43,44^

A PCR model was constructed by using the three PCs as predictors. Model significance was evaluated based on maximizing R^2^, minimizing the standard deviation, and requiring *p* < 0.01. Internal predictive ability was assessed by leave-one-out cross-validation (q^2^ > 0.5), and external validation was performed using the test set.^45^ All QSAR procedures were carried out with BuildQSAR^40^ and validated with STATISTICA 8.^41^ The applicability domain was characterized by using leverage values.^46^

SMILES strings derived from optimized structures were submitted to ADMETlab 3.0 ^47^ to estimate the pharmacokinetic properties and BBB penetration. Oral bioavailability was evaluated according to the Lipinski,^48^ Ghose,^49^ and Veber^50^ criteria. To quantitatively assess the CNS druglikeness of the selected compounds, the Pfizer CNS multiparameter optimization (CNS MPO) desirability score was calculated.^51^

Docking simulations were performed with AutoDock Vina 1.2.5 ^52,53^ with the BACE1 crystal structure (PDB: 4GID, chain A).^54^ The receptor was prepared with AutoDockTools.^53^ To accurately represent the binding environment under physiological conditions (pH 7.4), the protonation states of both the protein and ligands were carefully assigned. For the protein, the Protonate3D module within the Molecular Operating Environment (MOE)^55^ was used. The catalytic aspartates (ASP32 and ASP228) were explicitly assigned a charged (deprotonated) state, corresponding to their biologically active forms during catalysis. All other ionizable residues were protonated according to their predicted p*K*_a_ values at pH 7.4. For the ligands, the most probable protonation state at pH 7.4 was determined using the Chemicalize platform, and the resulting protonation forms were used as inputs for AutoDock Tools.^53^ This dual-protonation strategy ensures that the electrostatic complementarity captured during docking closely reflects biological interactions.

A 27 × 27 × 27 Å grid box was defined. The exhaustiveness parameter, which controls the global search effort, was set to 25. This value was selected based on recent benchmark studies showing that an exhaustiveness value of 25 provides improved docking performance and pose-prediction accuracy for large, flexible binding sites compared with the default value of 8, while requiring only a moderate increase in computational time. For each compound, 100 independent docking runs were performed. Cconvergence criteria consisted of a maximum number of evaluation steps (max_evals = 10^7^) and a root-mean-square deviation (RMSD) tolerance of 0.5 Å for termination of the local optimization. The resulting poses (5 runs × 20 poses) were clustered using a 2.0 Å RMSD tolerance, and the lowest-energy conformation from the most populated cluster was selected as the representative binding pose and visualized using Discovery Studio Visualizer.^56^

## 3 Results and Discussion

### 3.1 QSAR Model of BACE1 Inhibitors

The QSAR models were developed using MESP-derived molecular descriptors. The compound series was divided into training and test sets using k-means clustering (KM-CA). Figure 2 shows the dendrogram based on Euclidean distances; compound 37 appears as a potential outlier because of its greater distance from the remaining compounds.

**Figure 2:**
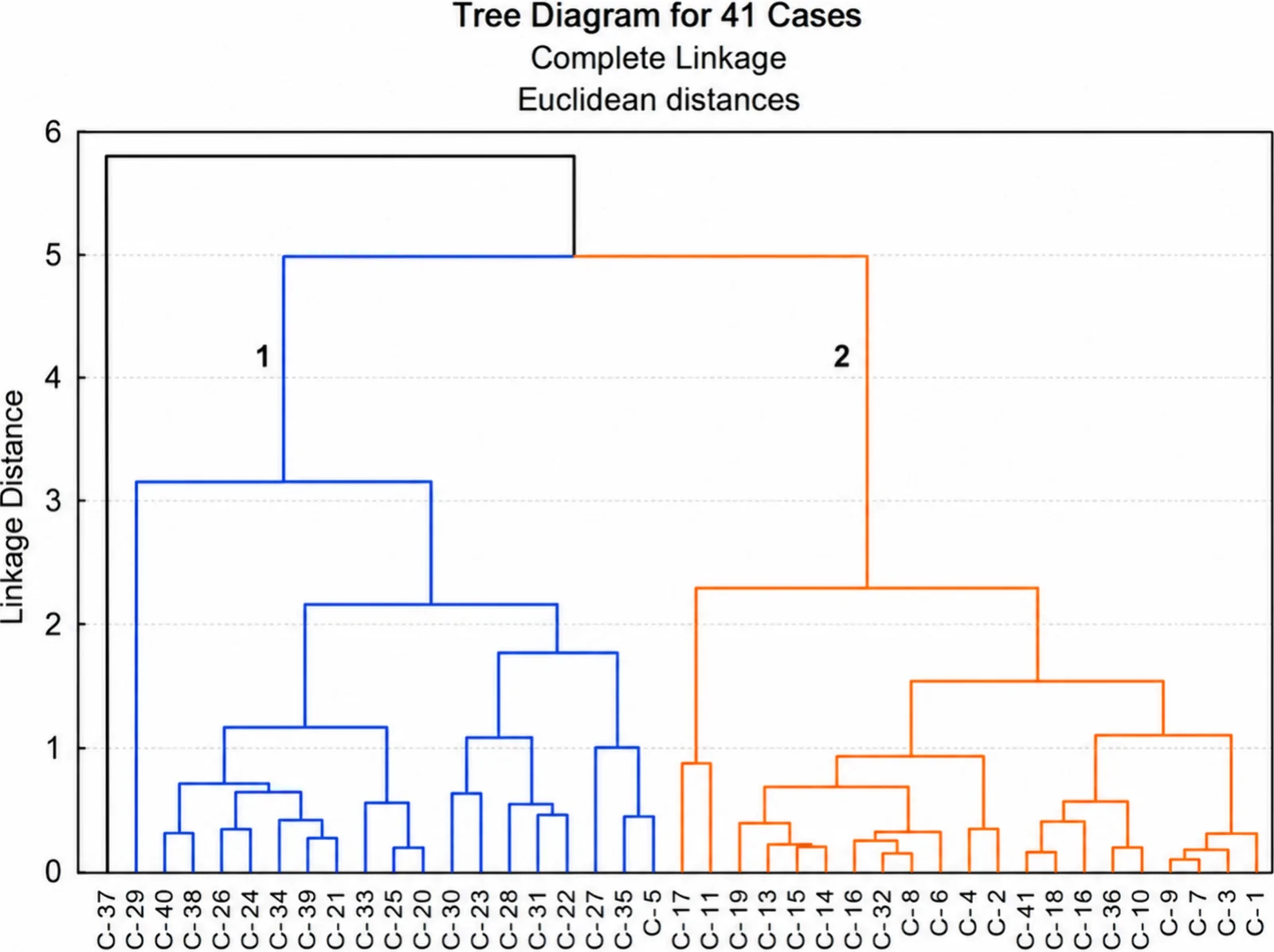
Dendrogram from hierarchical cluster analysis of the 41 BACE1 inhibitors. The *x*-axis indicates the individual compounds (numbered as in Table 1). The *y*-axis represents the Euclidean distance between compounds (complete linkage). Compound 37 (black) is a potential outlier due to its greater distance from all other compounds. Compounds in blue belong to Cluster 1, and those in red belong to Cluster 2.

#### 3.1.1 Principal Component Analysis of MESP Descriptors

Table 2 shows variable loadings for PC1 to PC6, which together accounted for 96% of the total variance. PC1 explains 33.8% of the variance, PC2 explains 25.2%, and PC3 explains 17.4%. Together, the first three principal components account for 76.4% of the total variance and were therefore retained for subsequent PCR modeling.

**Table 2:** Principal Component Loadings for MESP-Derived Molecular Descriptors Computed in This Work^a^

Molecular Descriptor	PC1	PC2	PC3	PC4	PC5	PC6
Total area, *A*						
Volume, *Vol*						
MESP max, *V*_*M*_						
MESP min, *V*_*m*_						
*Nmax*			**0.73**		**0.56**	
*Nmin*			**-0.53**		**0.62**	
*δx*						
*δy*						
*δz*						**-0.64**
Positive area, *A* ^+^				**-0.64**		
Negative area, *A* ^–^						
MESP mean, *V̅*	**0.70**					
POSITIVE MESP mean, $\overset{®}{V^{+}\;}$						
NEGATIVE MESP mean, $\overset{®}{V^{-}\;}$						
MESP variance, σ^2^						
Positive MESP variance, (σ^+^)^2^						
Negative MESP variance, (σ^–^)^2^		**-0.59**				
MESP deviation, Π						
ν parameter						
**Explained Variance**	**33.83**	**25.15**	**17.39**	8.41	7.15	4.04

a Loadings between −0.5 and 0.5 (−0.5 ≤ loading ≤ 0.5) are omitted to clarify the relationships among molecular descriptors and highlight their factor structure. PC1–PC3 explain 33.8%, 25.2%, and 17.4% of the variance, respectively (cumulative 76.4%). PC1–PC6 together explain 96.0% of the total variance. PC: principal component.

PC1 exhibits a high positive loading (0.70) for the mean of MESP. This indicates that a higher average electrostatic potential correlates with noncovalent interactions with charged groups in the BACE1 active site.

In PC2, the negative MESP variance (σ^–^)^2^ has a significant negative loading (−0.59). This indicates that heterogeneity in the distribution of negative electrostatic potential, associated with aromatic rings and electronegative groups, may enhance hydrogen-bonding or dipole–dipole interactions with the enzyme.

In PC3, Nmax (number of local MESP maxima) has a high positive loading (0.73), while Nmin (number of local MESP minima) exhibits a moderate negative loading (−0.53). These topological descriptors are associated with regions susceptible to nucleophilic and electrophilic interactions, respectively, and play an important role in specific protein–ligand interactions. The complete list of MESP descriptor values (19 descriptors) for all 41 compounds is provided in Table S1 of the Supporting Information.

These statistical correlations derived from PCA do not imply direct causation; experimental validation (e.g., site-directed mutagenesis or isothermal titration calorimetry) would be required to confirm the proposed electrostatic mechanisms.

#### 3.1.2 Cluster Analysis and Data Set Split

The dendrogram (Figure 2) reveals two main clusters, confirming structural diversity of the data set. Compound 37 appears at a greater distance from the remaining compounds, indicating that it may represent a structural outlier. KM-CA was used to divide the data set into a training set comprising 33 compounds (80%) and an external test set comprising 8 compounds (20%). The assignment of each compound to the training or test set is provided in Table S2.

#### 3.1.3 PCR QSAR Model for Biological Activity

The PCR model using PCs 1–3 yielded Equation 1:

$$\begin{array}{l} {\mathrm{pIC}}_{50}=+0.19\left(\pm 0.03\right)\mathrm{PC}1+0.08\left(\pm 0.04\right)\mathrm{PC}2-0.13\left(\pm 0.05\right)\mathrm{PC}3+8.26\left(\pm 0.08\right)\\ \mathrm{Training set}\;\left(n=33\right):R=0.92,R^{2}=0.85,s=0.23,F=53.13,p<0.0001,q^{2}=0.8\\ \mathrm{Test set}\;\left(n=8\right):R=0.95,s=0.19,F=13.1 \end{array}$$ 1

The principal component scores (PC1–PC3) for each compound are listed in Table S2.

The high values of *R* ^2^ (0.85) and *q* ^2^ (0.8) indicate good model fit and strong internal predictive ability, with no evidence of overfitting. The high correlation obtained for the external test set (R = 0.95) further supports the predictive capability of the model. In addition, a Y-randomization test involving 20 permutations yielded an average *R* ^2^ value below 0.2, indicating that the observed correlations are unlikely to have arisen by chance.

#### 3.1.4 Applicability Domain

The AD was defined using a Williams plot (Figure 3) based on leverage values (*h*_i_) and standardized residuals. The critical leverage threshold was calculated as

$$h^{*}=3p/n=3\times 3/41=0.29$$

where *p* is the number of PCs, and *n* is the number of compounds in the data set. Compounds with leverage values exceeding h* are considered to lie in regions of structural extrapolation.^57^

**Figure 3:**
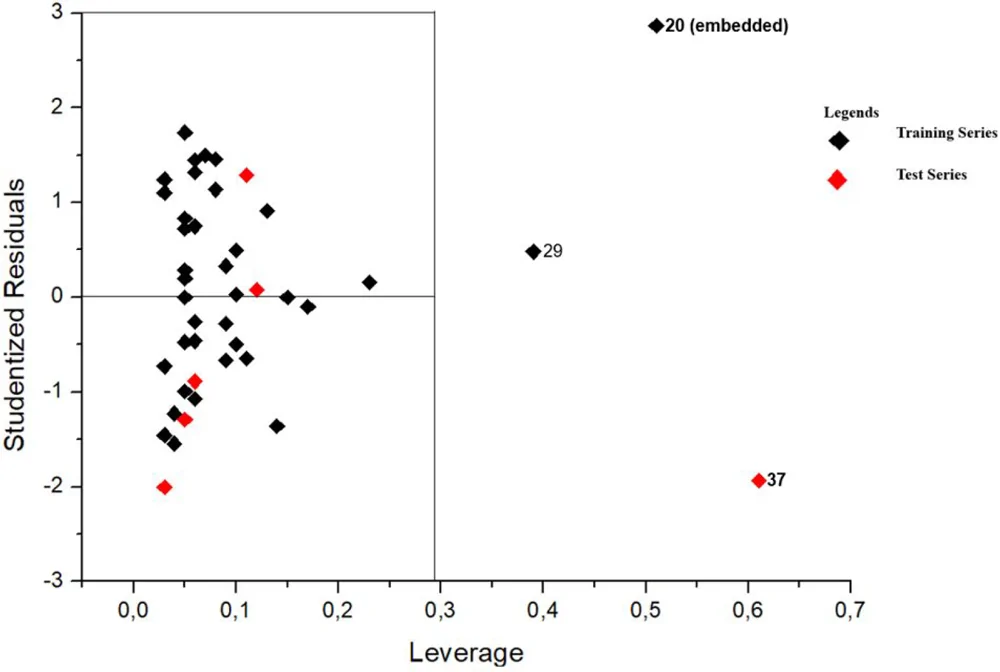
Williams plot for the PCR-QSAR model (eq 1). Standardized residuals (*y*-axis) are plotted against leverage values (*h*_i_, *x*-axis). The horizontal dashed lines mark ± 3 standard deviations (response outlier threshold). The vertical dashed line indicates the critical leverage h* = 3*p*/*n* = 0.29 (*p* = 3 PCs, *n* = 41). Black diamonds: training set; red diamonds: test set. Compounds 29 and 37 exceed h* (structural outliers) and have standardized residuals > 3 (response outliers), thus falling outside the applicability domain.

Compounds with standardized residual greater than 3 are classified as response outliers, whereas compounds with *h*_i_ > 0.29 are considered structural outliers. The leverage values and studentized residuals for all compounds are reported in Table S2.

Only compounds 29 and 37 exceed the critical leverage threshold (h* = 0.29). Compound 29 exhibits an extreme PC3 score (PC3 = 6.12) and the highest experimental activity in the data set (pIC_50_ = 9.70); whereas compound 37 has a PC1 score of 9.07, well outside the range observed for the training set (−3.74 to 3.11). Consequently, both compounds fall outside the applicability domain, and predictions for these molecules should be interpreted with caution.

### 3.2 Pharmacokinetic Properties, Drug-likeness, and Synthetic Accessibility

Lead candidates were required to simultaneously satisfy the following criteria: high BACE1 inhibitory activity (pIC_50_ > 8.0), BBB penetration probability > 0.2, low P-glycoprotein substrate probability (PgP_sub < 0.5), compliance with the Lipinski, Veber, and Ghose rules, synthetic accessibility (SA) < 5, and a CNS multiparameter optimization (CNS MPO) score ≥ 4.0.^58^

To contextualize the translational relevance of our pharmacokinetic filter, the predicted properties of N-[3-[(1S,5S,6S)-3-amino-1-(difluoromethyl)-5-(fluoromethyl)-2-thia-4-azabicyclo[4.1.0]­hept-3-en-5-yl]-4-fluorophenyl]-5-chloropyrazine-2-carboxamide (compound 5) were compared with those of approved CNS-active drugs (donepezil, rivastigmine) and BACE1 inhibitors that failed in clinical trials (verubecestat, atabecestat). Donepezil (logP = 3.6, TPSA = 38.8 Å^2^)^59^ and rivastigmine exhibit optimal CNS penetration. In particular, rivastigmine displays a high effective permeability coefficient (Pe = 20.0 × 10^–6^ cm·s^–1^) in the PAMPA-BBB assay,^60,61^ classifying it as a CNS^+^ compound, i.e., a molecule predicted to cross the BBB by passive diffusion. Compounds with Pe values below the established threshold (typically <4.39 × 10^–6^ cm·s^–1^) are classified as CNS^–^, indicating low brain penetration.

In our study, compound 5 (logP = 3.33, TPSA = 118.6 Å^2^, BBB = 0.44, CNS MPO = 4.0) exhibits a permeability profile comparable to that of donepezil, despite its slightly higher TPSA, which appears to be compensated by its very low probability of acting as a P-glycoprotein substrate (PgP_sub = 0.0047). In contrast, verubecestat and atabecestat exhibited poor BBB penetration and high hepatotoxicity, respectively, highlighting the value of the integrated pharmacokinetic filter employed in this work.^62^

Potential metabolic liabilities (e.g., O-dealkylation, N-demethylation, thioamide-associated toxicity) were identified for several compounds. Table S3 provides a detailed breakdown of each type of liability and the specific compounds affected.

After applying the filters to the 41 BACE1 inhibitors and the reference compound, *N*-[(2S)-1-({(2S.3R)-3-hydroxy-1-[(2-methylpropyl) amino]-1-oxobutan-2-yl} amino)-3-phenylpropan-2-yl]-5-[methyl­(methylsulfonyl)­amino]-*N*'-[(1R)-1-phenylethyl]­benzene-1.3-dicarboxamide (OGH), using ADMETlab 3.0, only compound 5 satisfied all criteria: pIC_50_ = 8.80, BBB = 0.44, PgP_sub = 0.0047, MW = 459.85, CLogP = 3.33, TPSA = 118.6 Å^2^, HBD = 2, no Lipinski violations, SA = 4.55, CNS MPO = 4.0. Benchmarking against approved CNS drugs confirms that compound 5 satisfies the principal CNS drug-likeness criteria (Lipinski, Veber, and Ghose rules) with a quantitative estimate of drug-likeness (QED) score of 0.66. The complete ADMET profiles, including MW, logP, TPSA, BBB, PgP_sub, Lipinski violations, CNS MPO, and synthetic accessibility, for all 41 compounds and OGH are provided in Table S4.

Recent studies have identified metabolic and cerebral perfusion alterations as potential biomarkers of Alzheimer’s disease progression,^63^ highlighting the importance of early BACE1 inhibition to prevent or mitigate such alterations. In addition, innovative CNS drug delivery strategies, such as those developed by Li and co-workers,^64^ may complement the design of BACE1 inhibitors with suboptimal permeability profiles.

The *N*-[3-[(7R)-9-amino-2,7-dimethyl-5,5-dioxo-5lambda6-thia-2,8-diazaspiro­[3.5]­non-8-en-7-yl]-4-fluorophenyl]-5-chloropyridine-2-carboxamide (compound 20) emerged as a secondary candidate (pIC_50_ = 8.21, BBB = 0.80, and PgP_sub = 0.0017), although its relatively low lipophilicity (CLogP = 1.86) may limit passive BBB diffusion. All other compounds, including the reference OGH (BBB = 0.0012), failed to satisfy the integrated pharmacokinetic filters.

The synthetic accessibility values obtained for all compounds (SA = 3.76–5.21) were significantly more favorable than that calculated for OGH (SA = 7.31), with five compounds exhibiting SA values below 4.0.

### 3.3 Molecular Docking of BACE1 Inhibitors

The molecular docking analysis revealed distinct binding profiles for the reference compound OGH and lead compound 5 (Figures 4 and 5). The reference compound exhibited a higher binding affinity (Δ*G* = −12.0 kcal/mol), supported by numerous polar interactions, including eight conventional hydrogen bonds involving THR120, GLN121, and ASP276, complemented by stacking interactions (π–π T-shaped with TYR119) and hydrophobic contacts (alkyl and π-alkyl interactions) (Table 3). However, this highly polar interaction profile is consistent with low membrane permeability and predicts an inability to cross the BBB, thereby limiting its potential as a BACE1 inhibitor for central nervous system applications.

**Figure 4:**
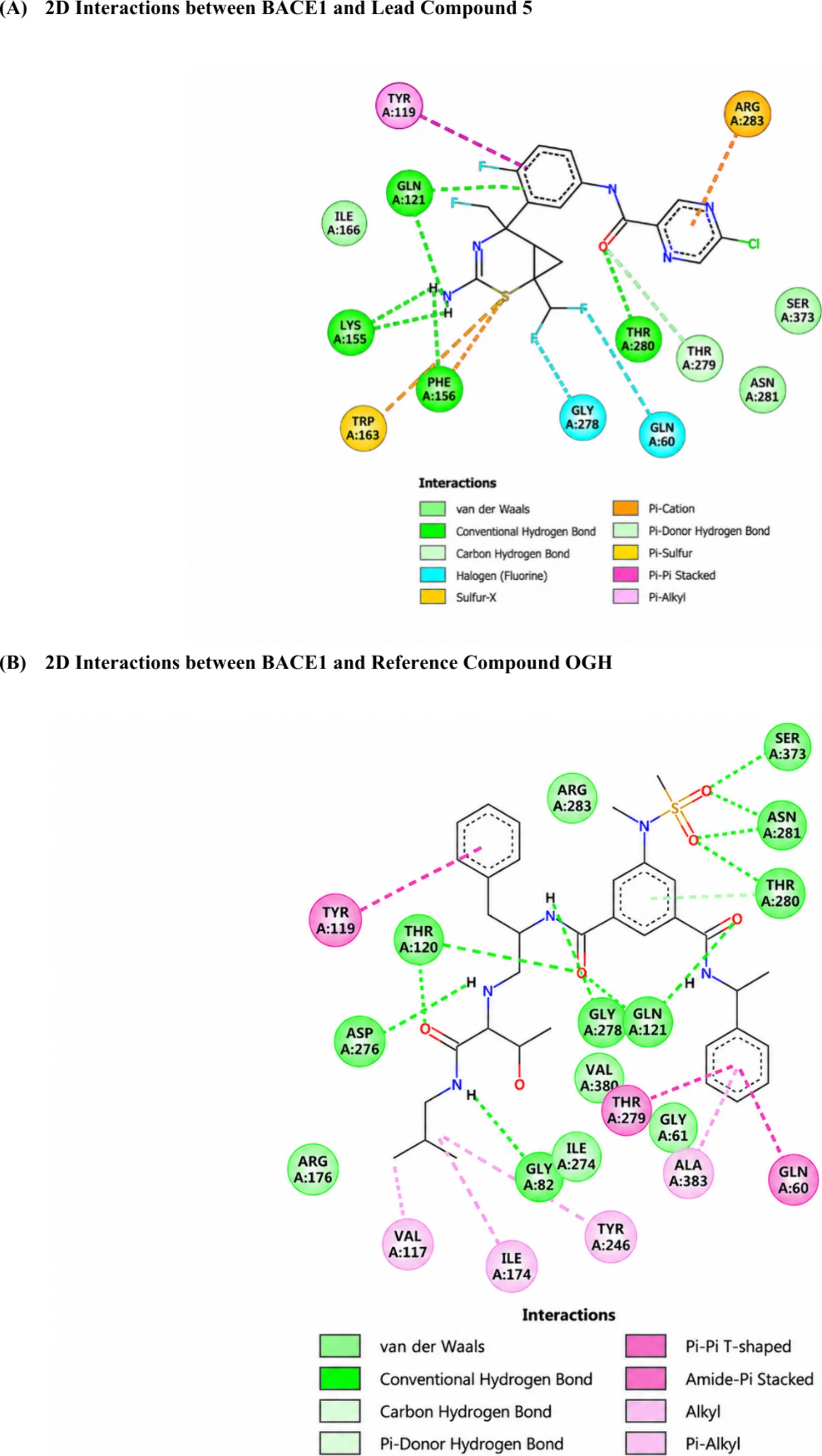
Two-dimensional interaction maps of (A) lead compound 5 and (B) reference compound OGH with the BACE1 active site (PDB: 4GID, chain A). All interactions are shown as dashed lines; the color code for each interaction type is displayed within the figure panels. Residues are labeled according to the BACE1 sequence. The structures of the compounds are given in Table 1.

**Figure 5:**
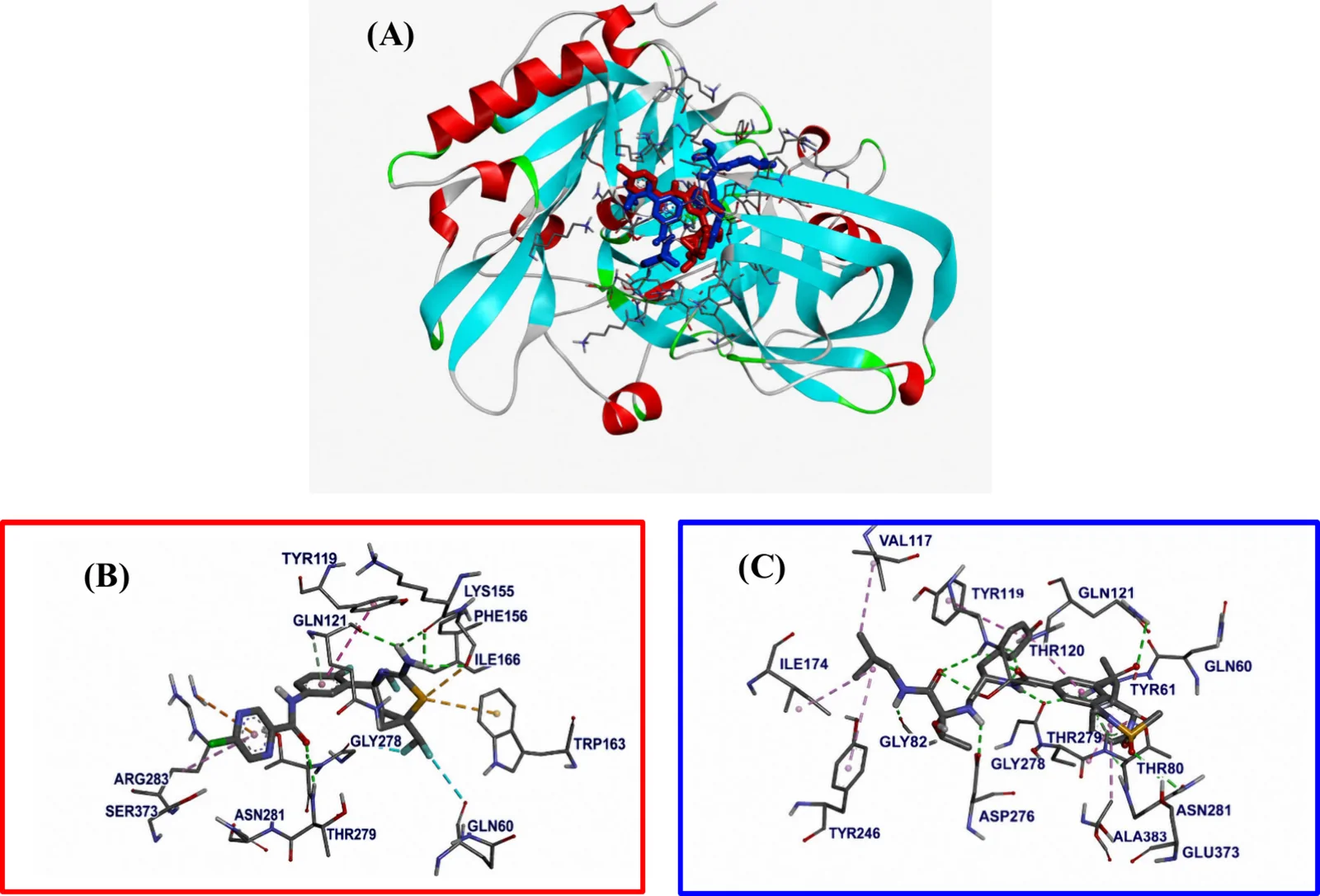
Three-dimensional representations of the docked complexes. (A) Overlay of reference compound OGH (blue) and lead compound 5 (red) in the BACE1 active site (gray surface). (B) Detailed view of compound 5 (red border) with key interacting residues. (C) Detailed view of OGH (blue border) with its hydrogen bond network. Noncovalent interactions are shown as dashed lines with the same color code described in Figure 4. Residues are labeled according to the BACE1 numbering (PDB: 4GID, chain A).

**Table 3:** Binding Affinity, Ligand Efficiency, and Interactions of Reference Compound OGH with BACE1^a^

Ligand	Binding affinity, Δ*G* (kcal/mol)	Ligand Efficiency	Protein Residue(s)	Distance (Å)	Chemical Interaction
OGH	–12.0	–0.26	THR120	2.79	Conventional Hydrogen Bond
			THR120	2.24	Conventional Hydrogen Bond
			GLN121	2.04	Conventional Hydrogen Bond
			ASN281	1.91	Conventional Hydrogen Bond
			SER373	2.50	Conventional Hydrogen Bond
			GLY278	2.24	Conventional Hydrogen Bond
			ASP276	2.49	Conventional Hydrogen Bond
			GLY278	2.15	Conventional Hydrogen Bond
			THR120	3.16	Carbon Hydrogen Bond
			THR280	3.16	π-Donor Hydrogen Bond
			TYR119	4.80	π-π T-shaped
			GLN60	4.12	Amide-π Stacked
			THR279	4.45	Amide-π Stacked
			ILE174	5.15	Alkyl
			VAL117	5.04	Alkyl
			TYR246	5.41	π-Alkyl
			ALA383	4.46	π-Alkyl

a Δ*G*: binding free energy (kcal/mol). LE: ligand efficiency, calculated as LE = −Δ*G*/NHA, where NHA is the number of heavy atoms. Interaction distances are given in Ångström (Å). Interaction types are defined in the legend of Figure 4.

In contrast, compound 5, selected as the lead candidate, exhibits favorable pharmacokinetic properties for CNS access. Although it displayed a lower but still significant binding affinity (Δ*G* = −9.0 kcal/mol), it also showed a slightly higher ligand efficiency (LE = 0.30) than the reference compound OGH (LE = 0.26). Ligand efficiency normalizes the binding affinity (Δ*G*) with respect to molecular size, allowing meaningful comparison among compounds of different complexity. It is defined as LE = −Δ*G*/NHA, where NHA is the number of heavy (non-hydrogen) atoms. An LE value ≥ 0.3 kcal mol^–1^ atom^–1^ is generally considered indicative of an efficient ligand that achieves substantial affinity without excessive molecular size.^65^

The binding mode of compound 5 is diverse and balanced, combining five conventional hydrogen bonds (with THR280, GLN121, LYS155 twice, and PHE156), halogen bonds (with GLN60 and GLY278), and a significant hydrophobic component, including π-cation (ARG283), π-sulfur (TRP163), and π-alkyl (ARG283) interactions (Table 4). Notably, compound 5 does not interact directly with the catalytic dyad, a feature that may contribute to its favorable balance between binding affinity and permeability.

**Table 4:** Binding Affinity, Ligand Efficiency, and Interactions of Lead Compound 5 with the BACE1 Receptor^a^

Ligand	Binding affinity, Δ*G* (kcal/mol)	Ligand Efficiency	Protein Residue(s)	Distance (Å)	Chemical Interaction
Compound 5	–9.0	–0.30	THR280	2.39	Conventional Hydrogen Bond
			GLN121	2.44	Conventional Hydrogen Bond
			LYS155	2.53	Conventional Hydrogen Bond
			LYS155	2.52	Conventional Hydrogen Bond
			PHE156	2.34	Conventional Hydrogen Bond
			THR279	3.43	Carbon Hydrogen Bond
			GLN60	3.24	Halogen (Fluorine)
			GLY278	3.11	Halogen (Fluorine)
			PHE156	3.17	Sulfur-X
			ARG283	4.84	π-Cation
			GLN121	3.26	π-Donor Hydrogen Bond
			TRP163	4.79	π-Sulfur
			TYR119	5.86	π-π Stacked
			ARG283	5.25	π-Alkyl

a Δ*G*: binding free energy (kcal/mol). LE: ligan d efficiency, calculated as LE = −Δ*G*/NHA, where NHA is the number of heavy atoms. Interaction distances are given in Ångström (Å). Interaction types are defined in the legend of Figure 4.

To further contextualize the binding mode of compound 5 and its potential translational relevance, a comparison was made with selected BACE1 inhibitors that reached advanced clinical trials but were subsequently discontinued.^62^ Table 5 summarizes the clinical status, affinity, key active-site interactions, causes of failure, and BBB penetration characteristics of three representative compounds (verubecestat, atabecestat, lanabecestat) based on the review by Patel et al.^62^ As shown in Table 5, these three BACE1 inhibitors rely on extensive direct interactions with the catalytic dyad (Asp32/Asp228). Such binding modes have been proposed to contribute to mechanism-based adverse effects, including cognitive worsening and hepatotoxicity as well as limited efficacy when administered to symptomatic patients.

**Table 5:** Comparison of Compound 5 with Selected Clinically Failed BACE1 Inhibitors^a^

Inhibitor (Code)	Clinical status (Phase)	BACE1 affinity (IC_50_/*K*_i_)	Key interactions with BACE1 active site	Main cause of failure/adverse effects	BBB penetration
Verubecestat (MK-8931)	Phase III (terminated)	IC_50_ = 2.2 nM	Interacts with catalytic dyad (Asp32/Asp228) via amidine moiety; occupies S3 pocket	Lack of efficacy; cognitive worsening; rashes; psychiatric events	Low (low P-gp efflux)
Atabecestat (JNJ-54861911)	Phase II/III (terminated)	IC_50_ = 9.8 nM	Brain-penetrable; interacts with catalytic aspartates	Elevation of liver enzymes (hepatotoxicity)	High (but hepatotoxic)
Lanabecestat (AZD3293)	Phase II/III (terminated)	*K*_i_ = 0.4 nM (BACE1)	Acyl guanidine class; interacts with S1 and S3 pockets	Psychiatric adverse events; weight loss; skin depigmentation	Moderate
OGH (reference)	Preclinical (in vitro)	Δ*G* = −12.0 kcal/mol (docking)	Eight H-bonds (THR120, GLN121, ASP276); π-π with TYR119; extensive polar interactions	Not applicable (no clinical trial)	Very low (BBB = 0.0012)
Compound 5 (lead)	Preclinical (*in silico* lead)	Δ*G* = −9.0 kcal/mol; pIC_50_ = 8.80	Occupies S1 (π-π with TYR119, H-bond with GLN121) and S3 (π-cation with ARG283); H-bonds (THR280, LYS155×2, PHE156); halogen bonds (GLN60, GLY278); π-sulfur with TRP163; no interaction with catalytic dyad	Not applicable (requires experimental validation)	Favorable (BBB = 0.44; PgP_sub = 0.0047; CNS MPO = 4.0)

a Data for verubecestat, atabecestat, and lanabecestat are based on the review by Patel et al. (2022).^62^ Binding affinities and interactions for OGH and compound 5 are from this study (docking and ADMETlab 3.0). BBB: blood-brain barrier; PgP_sub: P-glycoprotein substrate probability; CNS MPO: central nervous system multiparameter optimization score. Interaction types are defined in the legends of Figure 4.

In contrast, compound 5 combines high predicted potency (pIC_50_ = 8.80) and favorable BBB penetration (BBB = 0.44) without directly engaging the catalytic dyad. Instead, it strategically occupies the substrate-recognition pockets S1 and S3 through π-π stacking with TYR119, hydrogen bonding with GLN121, and a π-cation interaction with ARG283. This distinctive binding profile, characterized by balanced occupation of S1 and S3 pockets rather than direct interaction with the catalytic aspartates, may help avoid some of the toxicity and efficacy limitations associated with previous BACE1 inhibitors. Furthermore, the poor BBB penetration reported for verubecestat and the hepatotoxicity observed for atabecestat emphasize the importance of the integrated pharmacokinetic filter employed in this work, which identified compound 5 as a candidate combining potency, permeability, and favorable safety-related properties. These comparisons do not imply clinical readiness for compound 5 but rather its selection as an *in silico* lead deserving further experimental evaluation.

A more detailed structure–activity relationship (SAR) analysis was performed for compounds 5 and 6, which share the same N (pyridin 3 yl)­picolinamide core and differ only in the fluorination pattern of the distal aromatic ring (monofluorinated in 5 and difluorinated in 6). Despite their close structural similarity, compound 5 (pIC_50_ = 8.80; Δ*G* = −9.0 kcal/mol) is substantially more potent than compound 6 (pIC_50_ = 7.82; Δ*G* = −8.2 kcal/mol), corresponding to an approximately 10-fold difference in inhibitory activity (ΔΔ*G* ≈ 0.8 kcal/mol).

Table S5 summarizes the principal docking and activity differences between the two compounds. Docking simulations indicate that compound 6 fails to establish the extensive hydrogen-bonding network observed for compound 5. Specifically, it forms only one conventional hydrogen bond (with TYR119), and its two fluorine atoms participate in halogen-bond interactions with ARG176, TYR246, and PRO118, residues located farther from the catalytic dyad. The π–π interaction with TYR119 is retained but occurs at a shorter distance (4.49 Å versus 5.86 Å for compound 5), indicating a less favorable orientation that may reduce complex stability. The absence of key hydrogen bonds involving GLN121, LYS155, and THR280 correlates directly with its lower inhibitory activity of compound 6.

This experimental difference is quantitatively captured by the MESP-derived descriptors reported in Table S1. Compound 5 exhibits five MESP minima (Nmin = 5), whereas compound 6 has only two (Nmin = 2). Because *Nmin* has a negative loading on PC3 (−0.53) and PC3 has a negative coefficient in the PCR model (eq 1), a decrease in *Nmin* is associated with a reduction in the predicted pIC_50_, consistent with the observed decrease from 8.80 to 7.82. In addition, the negative MESP variance (σ^–^)^2^ is lower in compound 6 (1.45 × 10^–3^ vs 2.11 × 10^–3^ au^2^), indicating a more homogeneous distribution of negative electrostatic potential and, consequently, less pronounced electron-rich regions available for hydrogen-bond acceptance. These quantitative descriptors support the docking results and highlight the importance of precise fluorine substitution patterns for optimizing BACE1 inhibition. The introduction of a second fluorine without appropriate geometric complementarity appears to disrupt essential electrostatic and hydrogen-bonding interactions (see Table S1 for the complete descriptor comparison).

Overall, these interactions reflect a favorable balance between binding affinity and the pharmacokinetic properties required for BBB permeability, reinforcing the predicted ability of compound 5 to access the CNS. Taken together, these results identify compound 5, rather than the higher-affinity reference compound OGH, as the most promising candidate for the development of BACE1 inhibitors as it combines strong molecular binding with the essential pharmacokinetic requirement of BBB penetration.

### 3.4 Driving Forces for BACE1 Inhibition in Alzheimer’s Disease

#### 3.4.1 Conformational Dependence and Applicability Domain

The QSAR model was developed using gas-phase (isolated) conformations as a standard approach for the homogeneous calculation of molecular descriptors. However, the biologically relevant conformation is that adopted within the BACE1 active site. To assess the impact of conformational changes, isolated and embedded (docking-derived) conformations were compared for compounds 5 (Figure 6) and 20. MESP maps to the isolated and embedded conformations of compound 20 and OGH are shown in Figures S1 and S2, respectively.

**Figure 6:**
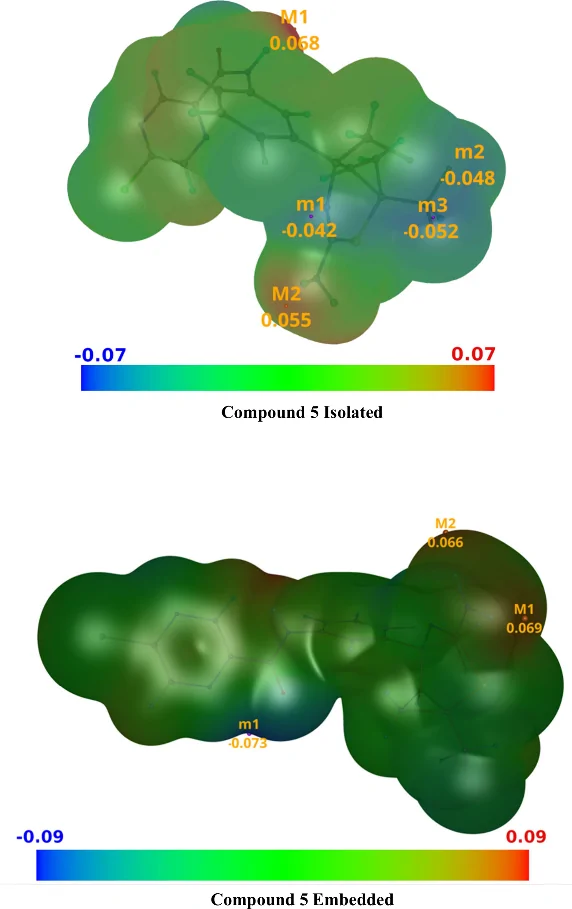
Molecular electrostatic potential (MESP) maps of compound 5. Top: isolated (gas-phase optimized) conformation; bottom: conformation extracted from the docking pose (embedded). The MESP was computed on the electron density isosurface (0.001 au). Blue regions indicate negative potential (electron-rich), red regions indicate positive potential (electron-deficient), and green indicates neutral. The positions of local MESP maxima (Nmax, red dots) and minima (Nmin, blue dots) are shown. The isolated conformation exhibits Nmin = 5 and Nmax = 4, while the embedded conformation shows a redistribution consistent with the binding pocket constraints.

For compound 5, predicted pIC_50_ changed from 8.39 (isolated conformation) to 8.32 (embedded conformation), well within the model error (residuals −0.48 and −0.41, respectively). In contrast, for compound 20, the embedded conformation yielded a predicted pIC_50_ of 5.28 compared with the experimental value of 8.21 (residual −2.93), well outside the acceptable prediction range. This behavior can be explained by the fact that compound 20 lies outside the AD (*h* = 0.51 > h* = 0.29; Figure 3). Thus, these results do not invalidate the model but rather highlight that predictions become unreliable when applied to conformations that deviate substantially from the structural space represented in the training set.

The observation that the embedded conformation of compound 20 falls outside the applicability domain, whereas compound 5 remains within an acceptable predictive range, underscores the importance of ligand conformational adaptability upon binding. This interpretation is supported by recent findings from Guo et al.,^66^ who demonstrated that phosphorylation at the T259 site of BACE1 significantly influences the conformational dynamics of the enzyme and its recognition of ligands and substrates. Their work provides a molecular rationale for understanding why certain inhibitors, such as compound 20, may adopt bioactive conformations that are poorly represented by gas-phase-optimized geometries, thereby limiting the predictive performance of QSAR models trained exclusively on isolated conformations. Furthermore, the authors showed that BACE1 conformational flexibility affects the binding orientation of small-molecule inhibitors, consistent with our observation that compound 5 maintains a more stable descriptor profile across conformations than does compound 20. Consequently, the incorporation of conformational dynamics data into QSAR workflows, as suggested by Guo et al., may further improve the predictive performance of future models for BACE1 inhibitors.

Therefore, the present QSAR model is suitable for the rapid virtual screening of isolated conformations within the AD. Embedded conformations are more appropriately used for mechanistic interpretation (e.g., mapping MESP extrema onto binding poses) than for quantitative prediction, unless the corresponding compound lies well within the AD.

#### 3.4.2 Protonation Effects

Our QSAR model used neutral, gas-phase optimized geometries, which is standard practice in descriptor-based QSAR studies. Most compounds in the data set, including compound 5, do not contain ionizable groups under physiological conditions. In contrast, compound 20 contains a tertiary amine, and docking calculations performed using its protonated form (pH 7.4) revealed an additional hydrogen bond with ASP228 that contributes to stabilization of the protein–ligand complex. Recalculation of MESP descriptors for protonated forms of all compounds in the data set would require substantial additional computational effort and was therefore beyond the scope of the present study. The molecular descriptors calculated from the neutral forms are reported in Table S1.

#### 3.4.3 Binding Mode and Driving Forces

Previous structural studies support the biological relevance of the interactions identified in this work. TYR119 and GLN121 belong to the S1 pocket, a critical region involved in APP substrate recognition.^67^ In particular, TYR119 participates in π-π stacking interactions with BACE1 inhibitors in high-resolution crystal structures (PDB: 4GID,^54^ 5HU0 ^68^), whereas GLN121 contributes to complex stabilization through directional hydrogen bonding. ARG283, located in the S3 pocket, interacts with positively charged ligands through π-cation interactions, thereby influencing inhibition selectivity and specificity.

As described in Section 3.3 and summarized in Table 4, compound 5 achieves high inhibitory activity through strategic occupation of the S1 and S3 pockets without engaging the catalytic dyad. Within the S1 pocket, it forms π-π stacking with TYR119 and a hydrogen bond with GLN121, whereas in the S3 pocket, it establishes π-cation and π-alkyl interactions with ARG283. These interactions are complemented by additional hydrogen bonds involving THR280 and LYS155, as well as halogen bonds with GLN60 and GLY278. Collectively, this interaction pattern provides a favorable balance between binding affinity and permeability, distinguishing compound 5 from inhibitors that rely primarily on direct catalytic-dyad interactions.

Recent findings by Wang and co-workers^69^ demonstrated that biased signaling through the cholecystokinin B receptor, particularly through the Gq pathway, may be beneficial in Alzheimer’s disease by promoting α-secretase activity and reducing amyloid-β pathology.^69^ Although BACE1 is an aspartyl protease rather than a G protein-coupled receptor, these findings emphasize the importance of pathway-selective pharmacology in Alzheimer’s disease. In a similar conceptual framework, the observation that compound 5 does not directly engage the catalytic dyad but instead occupies the substrate-recognition pockets S1 and S3 raises the possibility of a differentiated inhibition profile. Such a binding mode may potentially reduce some of the adverse effects associated with broad BACE1 inhibition, although this hypothesis requires experimental validation.

Notably, neither the reference compound OGH nor the lead compound 5 forms direct hydrogen bonds or other interactions with the catalytic residues ASP32 and ASP228 (Tables 3 and 4). However, OGH achieves its high binding affinity through an extensive network of polar interactions that is associated with poor predicted membrane permeability, whereas compound 5 balances affinity and permeability through strategic occupation of the S1 and S3 pockets.

The absence of direct interaction with catalytic aspartates (ASP32 and ASP228) appears to be compensated by steric and electronic occupation of the substrate recognition pockets. These results suggest that effective BACE1 inhibition may depend not only on direct catalytic dyad engagement but also on appropriate occupancy of the substrate recognition regions. Nevertheless, these conclusions are based on computational docking and QSAR correlations, and experimental validation (e.g., mutational studies or enzymatic assays) is required to confirm the proposed binding mechanism.

## 4 Conclusions

This study presents an integrated computational pipeline that combines QSAR modeling, ADMET filtering, and molecular docking for the rational design of BACE1 inhibitors targeting Alzheimer’s disease and identifies a promising computational lead candidate based on the *in silico* analyses. The MESP-based QSAR model exhibited robust predictive performance (R^2^ = 0.85, q^2^ = 0.8, and R_test = 0.95). PCA revealed that **PC1** (dominated by the mean MESP), **PC2** (dominated by the variance of the negative MESP), and **PC3** (dominated by *Nmax* and *Nmin*) capture key electrostatic and topological features associated with BACE1 inhibition. The applicability domain analysis identified compounds 29 and 37 as outside the reliable prediction space, thereby strengthening the confidence in the remaining model predictions.

Our integrated activity-pharmacokinetic filtering strategy, with particular emphasis on CNS accessibility, proved to be essential for translating computational predictions into a pharmacologically relevant context. Among all compounds evaluated, only N-[3-[(1S,5S,6S)-3-amino-1-(difluoromethyl)-5-(fluoromethyl)-2-thia-4-azabicyclo[4.1.0]­hept-3-en-5-yl]-4-fluorophenyl]-5-chloropyrazine-2-carboxamide (5) simultaneously satisfied the criteria for high inhibitory activity (pIC_50_ = 8.80), favorable BBB penetration (BBB = 0.44), drug-likeness, and synthetic accessibility (SA = 4.55).

Docking studies indicated that compound 5 preferentially occupies the S1 and S3 substrate-recognition pockets without directly engaging the catalytic dyad, thereby achieving a favorable balance between binding affinity and permeability. In particular, hydrogen-bonding involving THR280, GLN121, LYS155, and ARG283 together with a halogen-bond interaction appears to contribute to stabilization of the protein–ligand complex while avoiding direct contact with the catalytic aspartates ASP32 and ASP228. Consequently, compound 5 emerged as the most promising computational lead candidate identified in this study.

The predicted inhibitory profile of compound 5 appears to arise from strategic occupation of substrate recognition regions rather than direct catalytic dyad engagement, a finding that is consistent with the electrostatic features identified by the QSAR analysis. Nevertheless, all results presented herein are based on computational predictions, and experimental validation through enzymatic, cellular, and *in vivo* studies will be required before any translational conclusions can be drawn.

Overall, this work highlights the value of integrating QSAR, MESP-derived descriptors, ADMET analysis, and molecular docking to prioritize both potency and pharmacological developability, providing a rational framework for the discovery of CNS-active therapeutics for Alzheimer’s disease.

## Supplementary Material





## Notes

Additional tables (Table S1–Table S5) and figures (Figure S1 and Figure S2) (PDF)
Supplementary videos of MESP maps (ZIP)
