# Supplementary material for: Integrated Virtual Screening and Molecular Electrostatic Surface Potential Descriptors for Predicting BACE1 Inhibitor Interactions in Alzheimer’s Disease
Source: ACS Omega. 2026 Aug 7;11(32):47739–53. doi: 10.1021/acsomega.6c02969 (PMC13491998; doi:10.1021/acsomega.6c02969)
Supplement: Supplementary file 1 [file ao6c02969_si_001.pdf]

SUPPORTING INFORMATION

Integrated Virtual Screening and Molecular Electrostatic Surface Potential Descriptors for Predicting BACE1 Inhibitor Interactions in Alzheimer's Disease.

Yoanna Alvarez-Ginarte[1*], Rafael López[2*], José Manuel García de la Vega[2], Ignacio De Ema[2] and Daniel Alpízar-Pedraza[3].

[1]Universidad de la Habana, Facultad de Química, Havana, 10400, CU.

[2]**Universidad Autónoma de Madrid, Química Física Aplicada**, Madrid, 28049, Madrid, **ES**.

[3]Center for Pharmaceuticals Research and Development (CIDEM), 10400, La Habana, C**U.**

TABLE OF CONTENTS

Table S1. Experimental and calculated biological activities (pIC_{50}) and MESP-derived molecular descriptor values for the 41 BACE1 inhibitors included in the dataset.

Table S2. Experimental pIC_{50} values, principal component scores (PC1–PC3), leverage values, studentized residuals, and set assignment (training/test) for compounds 1–41.

Table S3. Metabolic hotspots identified in the 41 N-(pyridin-3-yl)picolinamide derivatives.

Table S4. Experimental pIC_{50} values and ADMETlab 3.0 pharmacokinetic properties for the 41 BACE1 inhibitors and the reference compound OGH.

Table S5. Comparative docking and activity data for compounds 5 and 6: structural basis for the effect of difluoro substitution on BACE1 inhibition.

Figure S1. Molecular electrostatic potential (MESP) maps of reference compound OGH in its isolated conformation and in the conformation, it adopts when binding to the BACE1 active site.

Figure S2. Molecular electrostatic potential (MESP) maps of compound 20 in its isolated conformation and in the conformation, it adopts when binding to the BACE1 active site.

Geometry Videos. Animations of three-dimensional computer graphics of MESP maps for the Compounds: 5. 20 and OGH in its isolated conformation and in the conformation it adopts when binding to the active site of the BACE 1 enzyme

Table S1. Experimental and calculated biological activities (pIC_{50}) and MESP-derived molecular descriptor values for the 41 BACE1 inhibitors included in the dataset

**No.**[a]	[b]BACE 1 pIC_{50}	[c]BACE 1 pIC_{50}	[d]$A\times10^{-3}$	[e]$Vol\times10^{-3}$	[f]MESP max	[g]MESP min
1	7.96	7.84	1.56	3.19	0.06	-0.06
2	7.92	8.22	1.35	2.69	0.06	-0.05
3	7.62	7.61	1.38	2.82	0.06	-0.07
4*	7.92	8.23	1.55	3.17	0.07	-0.05
**5 (isolated)**	8.80	8.39	1.45	2.97	0.07	-0.05
**5 (embedded)**	8.80	8.32	1.46	3.03	0.09	-0.07
6	7.82	8.18	1.42	2.87	0.07	-0.05
7	8.08	7.74	1.52	3.08	0.07	-0.06
8	8.25	7.98	1.49	3.01	0.07	-0.06
9*	8.21	7.91	1.53	3.13	0.06	-0.06
10	7.64	7.90	1.35	2.69	0.06	-0.06
11	7.31	7.46	1.36	2.71	0.07	-0.06
12	8.82	8.41	1.48	3.03	0.07	-0.06
13	7.92	8.10	1.54	3.17	0.06	-0.06
14	7.64	7.70	1.40	2.76	0.06	-0.06
15*	7.66	8.13	1.46	3.04	0.06	-0.06
16	7.88	8.23	1.48	2.95	0.06	-0.05
17	7.09	7.11	1.37	2.72	0.06	-0.06
18	7.92	7.85	1.43	2.79	0.07	-0.06
19	7.93	8.22	1.53	2.98	0.07	-0.06
20***(isolated)**	8.21	8.42	1.57	3.26	0.07	-0.06
20***(embedded)**	8.21	5.28	1.54	3.40	0.13	-0.06
21	8.52	8.52	1.51	3.19	0.08	-0.06
22	9.00	9.00	1.54	3.22	0.07	-0.05
23	9.00	9.07	1.68	3.32	0.06	-0.06
24	8.52	8.25	1.52	3.12	0.08	-0.06
25	8.52	8.17	1.55	3.28	0.07	-0.07
26	8.30	7.99	1.52	3.10	0.07	-0.06
27*	9.00	8.98	1.74	3.48	0.07	-0.06
28	8.70	8.86	1.66	3.43	0.07	-0.06
29	9.70	9.61	1.75	3.63	0.06	-0.06
30*	9.00	9.12	1.64	3.48	0.07	-0.06
31	9.22	8.88	1.64	3.45	0.07	-0.06
32*	8.04	8.15	1.48	3.02	0.07	-0.05
33	8.41	8.53	1.54	3.06	0.07	-0.07
34	8.77	8.56	1.43	2.83	0.08	-0.06
35	8.46	8.70	1.52	3.03	0.07	-0.05
36	7.68	7.64	1.45	2.89	0.06	-0.06
37*	7.96	8.25	1.55	3.09	0.06	-0.09
38	8.66	8.46	1.61	3.36	0.06	-0.06
39	8.01	7.93	1.50	3.03	0.05	-0.06
40	8.73	8.43	1.55	3.26	0.08	-0.06
41	8.03	7.86	1.41	2.82	0.07	-0.06
**OGH (isolated)**	10.82	10.90	2.53	5.54	0.06	-0.07
**OGH (embedded)**	10.82	10.73	2.54	5.42	0.06	-0.11

Table S1. Continuation.

**No.**[a]	[h]*kntmax*	[i]*kntmin*	[j]δx /10	[k]δy /10	[l] δz/10	[m]$A^{+}\times10^2$	[n]$A^{-}\times10^2$	[ñ]MESP mean $\overline{V}\times10^3$
1	6	2	2.16	2.53	2.82	9.49	6.10	4.57
2	5	3	2.96	1.98	1.68	7.48	5.98	2.87
3	3	3	2.14	2.86	1.50	8.15	5.68	2.15
4*	3	2	2.39	2.87	1.87	8.83	6.66	5.16
**5 (isolated)**	**3**	**5**	2.39	2.69	1.77	8.83	5.71	**5.15**
**5 (embedded)**	**3**	**1**	1.90	2.66	2.67	7.92	6.64	**2.42**
6	3	2	2.62	2.41	1.56	8.43	5.77	5.57
7	3	4	2.40	2.79	2.05	9.52	5.69	4.76
8	7	2	2.53	2.88	1.73	8.54	6.34	5.98
9*	7	3	2.54	2.88	1.90	8.98	6.32	5.79
10	3	3	2.42	2.72	1.57	7.80	5.72	3.13
11	3	2	2.41	2.72	1.58	7.80	5.83	3.98
12	3	3	1.97	2.57	2.57	8.92	5.93	5.02
13	3	2	2.38	3.03	1.86	9.25	6.18	4.26
14	3	4	2.61	2.63	1.86	8.33	5.69	3.01
15*	3	3	2.42	2.66	1.70	9.27	5.32	5.53
16	3	3	1.63	2.63	2.84	8.81	5.98	4.70
17	3	2	2.69	2.62	1.74	7.55	6.17	1.69
18	3	3	2.52	2.41	2.01	8.69	5.60	4.11
19	4	3	2.61	2.47	2.20	9.45	5.83	5.42
20***(isolated)**	**3**	**4**	2.43	3.17	1.82	10.08	5.63	**3.85**
20***(embedded)**	**4**	**11**	2.03	2.65	2.65	7.07	8.36	**4.88**
21	3	3	2.30	2.22	2.70	9.40	5.67	4.92
22	3	6	2.36	2.78	1.81	10.42	4.93	7.13
23	6	4	2.16	2.73	2.56	11.07	5.70	5.92
24	3	3	2.49	2.74	1.83	9.68	5.47	3.57
25	3	2	2.40	2.96	1.59	9.92	5.61	3.82
26	3	2	2.30	2.93	1.67	9.41	5.76	3.50
27*	3	3	2.65	3.32	1.96	10.55	6.81	6.26
28	3	4	2.43	2.16	2.90	10.44	6.11	3.30
29	3	4	1.84	2.85	3.44	12.39	5.14	5.36
30*	3	4	2.81	2.79	2.43	11.27	5.15	5.93
31	3	4	2.65	2.49	2.60	10.68	5.69	4.24
32*	3	4	2.30	2.99	1.62	8.80	6.04	4.68
33	4	3	2.12	2.17	3.00	9.98	5.45	4.87
34	3	4	2.11	2.16	2.79	9.52	4.73	7.27
35	3	4	2.02	2.27	2.88	9.90	5.28	6.91
36	3	1	2.01	1.91	3.25	8.41	6.04	2.36
37*	4	2	2.19	2.89	2.44	10.40	5.14	4.31
38	5	4	2.63	3.24	1.62	9.83	6.30	4.35
39	4	3	2.44	3.13	1.58	9.50	5.45	3.75
40	3	3	2.41	2.75	1.85	9.78	5.67	4.36
41	3	4	1.85	2.98	1.86	8.63	5.43	3.25
**OGH (isolated)**	9	6	-3.54	-2.91	-2.27	1.87	6.63	9.19
**OGH (embedded)**	4	4	-4.21	-2.53	-2.63	1.64	9.01	1.71

Table S1. Continuation.

**No.**[a]	[o] $\overline{V^{+}}\times10^{2}$	[p] $\overline{V^{-}}\times10^{2}$	[q] $\sigma^2\times10^3$	[r] $(\sigma^+)^2\times10^3$	[s] $(\sigma^-)^2\times10^3$	[t] $\Pi\times10^2$	[u] $\nu\times10$
1	1.69	-1.46	3.34	1.57	1.76	1.54	0.25
2	1.97	-1.82	3.63	1.60	2.03	1.88	0.25
3	1.82	-2.09	4.29	1.27	3.02	1.90	0.21
4*	1.97	-1.40	3.22	1.79	1.43	1.69	0.25
**5 (isolated)**	2.10	-1.94	3.74	1.63	**2.11**	1.96	0.25
**5 (embedded)**	2.04	-1.90	5.24	2.75	**2.48**	1.97	0.25
6	2.06	-1.64	3.55	2.10	1.45	1.84	0.24
7	2.05	-2.15	3.82	1.63	2.19	1.99	0.24
8	2.32	-1.72	4.10	2.37	1.73	2.03	0.24
9*	2.41	-2.03	3.86	2.08	1.78	2.19	0.25
10	2.20	-2.26	3.70	1.65	2.05	2.18	0.25
11	2.26	-2.10	3.57	1.76	1.81	2.15	0.25
12	2.24	-2.12	3.60	1.59	2.01	2.12	0.25
13	2.01	-1.95	3.91	1.61	2.30	1.93	0.24
14	2.03	-2.24	3.93	1.64	2.29	2.07	0.24
15*	2.08	-2.10	3.72	1.49	2.23	1.97	0.24
16	2.19	-2.07	3.43	1.59	1.84	2.07	0.25
17	1.87	-1.91	4.40	1.57	2.83	1.87	0.23
18	2.03	-2.11	4.14	1.87	2.27	2.00	0.25
19	2.07	-1.93	4.24	2.24	2.01	1.94	0.25
20***(isolated)**	1.87	-2.27	5.19	1.49	**3.71**	1.93	0.20
20***(embedded)**	4.22	-2.67	11.98	9.78	**2.20**	3.44	0.15
21	2.23	-2.40	4.82	1.88	2.94	2.20	0.24
22	2.34	-2.73	4.65	1.87	2.78	2.26	0.24
23	2.16	-2.46	4.29	1.68	2.61	2.13	0.24
24	1.82	-2.24	4.93	1.85	3.08	1.89	0.23
25	1.81	-2.15	5.26	1.53	3.73	1.85	0.21
26	1.86	-2.12	4.70	1.44	3.26	1.89	0.21
27*	2.37	-2.08	3.52	1.86	1.66	2.16	0.25
28	1.93	-2.41	3.95	1.46	2.49	2.04	0.23
29	1.70	-2.27	3.38	1.03	2.35	1.68	0.21
30*	2.02	-2.53	4.91	1.71	3.19	1.99	0.23
31	1.99	-2.52	4.33	1.34	2.99	2.06	0.21
32*	2.09	-1.89	3.89	1.95	1.94	1.96	0.25
33	2.08	-2.43	5.31	2.01	3.30	2.10	0.24
34	2.30	-2.43	5.13	2.34	2.78	2.16	0.25
35	2.16	-2.06	3.92	1.67	2.25	1.97	0.24
36	1.64	-1.71	3.26	1.17	2.09	1.64	0.23
37*	2.22	-3.20	7.96	1.26	6.70	2.42	0.13
38	2.29	-2.46	4.56	1.64	2.92	2.28	0.23
39	1.86	-2.21	3.99	1.12	2.87	1.90	0.20
40	2.01	-2.28	4.64	1.95	2.69	2.02	0.24
41	1.78	-1.99	3.75	1.56	2.20	1.80	0.24
**OGH (isolated)**	2.22	-2.75	0.49	1.21	3.76	2.00	0.18
**OGH (embedded)**	1.76	-2.74	0.72	1.26	5.96	2.06	0.14

[a]No. of compounds in the study. chemicals marked with an asterisk in the test set. [b]Experimental Negative Logarithm of the IC_{50} BACE 1 value in Molar (M). [c]Calculated Logarithm of the IC_{50} BACE 1 value in Molar (M). [d]Area of density isosurface in bohr^2. [e]Volume enclosed by the density isosurface in bohr^3. [f]Highest value of MESP on the density isosurface. [g]Lowest value of MESP on the density isosurface. [h]Number of disjoint regions with maxima. [i]Number of disjoint regions with minima. [j,k,l]Dimensions of the surrounding box. [m]Area of density isosurface with positive MESP values. in bohr^2. [n]Area of density isosurface with negative MESP values. in bohr^2. [ñ]Average value of MESP on isosurface (Ω) in au (Hartree/e). [o]Average value of MESP on density isosurface with positive MESP (Ω^+) in au. [p]Average value of MESP on density isosurface with negative MESP (Ω^-) in au. [q]Variance of MESP on isosurface in au^2. [r]Variance of MESP on isosurface with positive MESP values in au^2. [s]Variance of MESP on isosurface with negative MESP values in au^2. [t]Mean of MESP deviations (sum of absolute differences between point and mean values) in au (Hartree/e). [u]$\nu = (\sigma^+)^2 (\sigma^-)^2 / (\sigma^2)^2$. Chemicals marked with asterisk were selected in test set.

Table S2. Experimental pIC_{50} values, principal component scores (PC1–PC3), leverage values, studentized residuals, and set assignment (training/test) for compounds 1–41.

No.	pIC50	PC1	PC2	PC3	Leverage	Studentized Residuals	Set
1	7.96	-3.74	0.557	0.70	0.10	0.499	training
2	7.92	-2.71	1.505	2.79	0.14	-1.358	training
3	7.62	-3.04	2.131	0.18	0.10	0.028	training
4	7.92	-1.96	-1.083	0.31	0.05	-1.291	test
5 (isolated)	8.80	-0.02	-2.372	0.14	0.06	0.750	training
5(embedded)	8.80	-0.42	-1.377	1.587	0.05	0.201	training
6	7.82	-1.23	-1.012	-0.36	0.04	-1.543	training
7	8.08	-2.69	0.532	-0.59	0.06	1.450	training
8	8.25	-1.69	-1.541	-1.54	0.08	1.136	training
9	8.21	-1.99	-1.945	-2.02	0.11	1.290	test
10	7.64	-1.86	-0.716	-1.27	0.06	-1.074	training
11	7.31	-1.65	0.563	-2.79	0.11	-0.648	training
12	8.82	-0.65	0.395	1.56	0.05	1.741	training
13	7.92	-1.01	0.607	0.25	0.03	-0.729	training
14	7.64	-1.18	0.681	-1.72	0.06	-0.256	training
15	7.66	-0.53	0.062	-0.33	0.03	-2.007	test
16	7.88	-1.20	-0.098	0.54	0.03	-1.455	training
17	7.09	-1.86	3.465	-2.32	0.17	-0.103	training
18	7.92	-1.31	-0.023	-1.36	0.05	0.286	training
19	7.93	-1.06	-1.314	-0.50	0.04	-1.227	training
20 (isolated)	8.21	2.43	1.245	0.14	0.06	-0.888	test
20(embedded)	8.21	-13,66	-4,75	0,02	0.51	2.866	test
21	8.52	1.38	-1.407	-0.63	0.05	0.001	training
22	9.00	2.66	-3.564	-0.55	0.15	0.002	training
23	9.00	1.93	-1.895	1.58	0.09	-0.278	training
24	8.52	0.56	0.186	-0.30	0.03	1.105	training
25	8.52	1.95	2.166	-0.18	0.07	1.496	training
26	8.30	0.67	2.194	-0.27	0.06	1.315	training
27	9.00	0.19	-3.423	1.15	0.12	0.079	test
28	8.70	1.13	0.088	2.51	0.09	-0.667	training
29	9.70	2.45	0.685	6.12	0.39	0.481	training
30	9.00	3.11	-1.562	1.30	0.10	-0.495	test
31	9.22	2.46	0.122	1.76	0.08	1.455	training
32	8.04	-1.69	-1.452	-0.56	0.06	-0.461	training
33	8.41	2.08	-0.349	-0.28	0.05	-0.471	training
34	8.77	1.42	-3.064	-1.70	0.13	0.911	training
35	8.46	0.04	-1.454	1.21	0.05	-0.991	training
36	7.68	-2.61	4.552	1.90	0.23	0.161	training
37	7.96	9.07	4.016	-3.24	0.61	-1.936	test
38	8.66	1.92	-0.857	-0.90	0.05	0.830	training
39	8.01	0.80	2.872	-0.16	0.09	0.330	training
40	8.73	0.77	-0.871	-0.28	0.03	1.244	training
41	8.03	-1.30	1.38	-0.27	0.05	0.722	training

PC1–PC3: principal component scores from the PCR-QSAR model. Leverage values (hi) and studentized residuals were used to define the applicability domain (see section 3.1.4). Compounds marked with an asterisk () belong to the test set.

Table S3. Metabolic hotspots identified in the 41 N-(pyridin-3-yl)picolinamide derivatives.

Type of liability	Typical metabolic fate	Affected compounds (numbers)
Methoxy (–OCH_3) or methoxymethyl (–CH_2OCH_3)	Rapid O-demethylation (CYP450)	7, 17, 36, 37, 39
Fluoromethoxy (–OCH_2F)	O-dealkylation releasing formaldehyde and fluoride	8, 9, 19, 38
N-methyl (amines, piperazines, imidazolones)	N-demethylation (CYP450)	20, 23, 24, 25, 26, 33
Thioamide (C(=S)–N)	Formation of reactive metabolites (toxicity risk)	32
Terminal alkyne (C≡C–H)	Oxidation to reactive ketene or epoxide	14
Cyclic ether (tetrahydrofuran, oxolane)	α-Hydroxylation → ring opening	2, 4, 22, 40
Difluoromethyl (–CHF_2) or trifluoromethyl (–CF_3)	Potential defluorination	1, 4, 5, 6, 8, 9, 13, 35
Cyanopyridine (C≡N on pyridine ring)	Slow hydrolysis to carboxamide or carboxylic acid	7, 10, 11, 12, 14, 15, 16, 18, 21, 22, 23, 27, 28, 29, 30, 31, 33, 34, 35, 39, 40

The table lists typical metabolic fates and the specific compounds (numbers as in Table 1) that contain each structural liability.

Table S4. Experimental pIC_{50} values and ADMETlab 3.0 pharmacokinetic properties for the 41 BACE1 inhibitors and the reference compound OGH.

No.	BACE 1 pIC_{50}	MW (Da)	Consensus Log P	nHBD	TPSA (Å²)	BBB	PgP_sub	Lipinski #violations
OGH	10.82	680.96	2.06	8	186.95	0.0012	0.067	3
1	7.96	513.80	4.10	2	102.49	0.54	0.38	1
2	7.92	390.41	2.45	2	114.90	0.093	0.58	0
3	7.62	406.86	2.88	2	114.90	0.27	0.13	0
4	7.92	504.45	4.03	2	114.90	0.98	0.19	1
**5**	**8.80**	**459.85**	**3.33**	**2**	**118.56**	**0.44**	**0.0047**	**0**
6	7.82	463.81	3.35	2	118.56	0.00	0.011	0
7	8.08	431.46	2.42	2	138.69	0.013	0.087	0
8	8.25	460.42	3.95	2	114.90	0.041	0.75	0
9	8.21	456.46	3.91	2	114.90	0.0036	0.72	0
10	7.64	389.33	2.37	2	113.39	0.063	0.033	0
11	7.31	407.32	2.35	2	113.39	0.00	0.044	0
12	8.82	429.40	2.82	2	113.39	0.14	0.15	0
13	7.92	488.38	3.60	2	98.83	0.06	0.88	0
14	7.64	375.36	1.90	2	113.39	0.00	0.0021	0
15	7.66	429.40	2.99	2	113.39	0.04	0.29	0
16	7.88	431.37	2.05	2	122.62	0.00	0.34	0
17	7.09	371.37	1.78	2	111.72	0.02	0.92	0
18	7.92	401.34	2.46	2	113.39	0.01	0.055	0
19	7.93	439.36	2.92	2	111.72	0.00	0.91	0
**20**	**8.21**	**465.93**	**1.86**	**2**	**126.13**	**0.80**	**0.0017**	**0**
21	8.52	439.49	1.44	2	155.91	0.01	0.0057	0
22	9.00	457.48	1.42	2	155.91	0.00	0.13	0
23	9.00	440.48	0.70	2	159.15	0.00	0.65	0
24	8.52	451.90	2.17	2	126.13	0.07	0.32	0
25	8.52	451.95	3.10	2	118.18	0.57	0.75	0
26	8.30	438.91	2.00	2	121.42	0.12	0.99	0
27	9.00	511.50	2.56	2	145.21	0.04	0.25	1
28	8.70	469.54	1.88	2	145.21	0.06	0.96	0
29	9.70	482.57	2.95	2	141.97	0.53	0.17	0
30	9.00	468.55	2.63	2	141.97	0.031	0.076	0
31	9.22	469.54	2.08	2	154.86	0.00	0.051	0
32	8.04	441.93	2.36	2	141.15	0.21	0.91	0
33	8.41	419.41	1.37	2	131.09	0.00	0.55	0
34	8.77	390.37	1.34	2	134.87	0.00	0.57	0
35	8.46	440.38	2.03	2	134.87	0.01	0.025	0
36	7.68	407.39	2.60	2	102.49	0.02	0.51	0
37	7.96	434.41	2.75	2	98.83	0.34	0.51	0
38	8.66	484.49	2.94	2	132.12	0.00	0.99	0
39	8.01	395.43	2.63	2	113.39	0.01	0.38	0
40	8.73	432.45	2.21	2	137.18	0.00	0.0056	0
41	8.03	394.38	2.40	2	106.15	0.07	0.78	0

Table S4. Continuation.

No.	Ghose #violations	Veber #violations	logD	QED	LM human	CNS MPO	Synthetic Accessibility
OGH	3	2	2.80	0.16	0.99	2.0	7.31
1	2	0	3.06	0.59	0.00	3.5	4.55
2	0	0	2.19	0.84	0.90	4.2	4.22
3	0	0	2.36	0.81	0.98	4.3	4.19
4	2	0	3.40	0.60	0.72	3.4	4.76
**5**	**0**	**0**	**3.04**	**0.66**	**0.05**	4.0	**4.55**
6	0	0	2.99	0.67	0.01	3.9	4.51
7	0	0	2.32	0.75	0.84	3.6	4.55
8	1	0	3.11	0.64	0.96	3.8	4.23
9	0	0	2.93	0.62	0.94	3.9	4.34
10	0	0	2.47	0.83	0.00	4.2	3.76
11	0	0	2.45	0.75	0.00	4.2	3.81
12	0	0	2.15	0.77	0.01	4.3	4.51
13	1	0	3.16	0.64	0.00	3.7	4.80
14	0	0	2.54	0.79	0.01	4.0	4.36
15	0	0	2.49	0.77	0.00	4.2	4.43
16	0	0	2.47	0.76	0.01	4.1	4.61
17	0	0	2.23	0.84	0.00	4.2	4.50
18	0	0	2.38	0.81	0.01	4.3	4.15
19	0	0	2.54	0.66	0.00	4.1	4.71
**20**	**0**	**0**	**2.19**	**0.71**	**0.00**	**3.6**	**4.58**
21	0	1	1.30	0.72	0.01	2.8	4.71
22	0	1	1.72	0.71	0.00	2.9	4.63
23	0	1	1.25	0.71	0.00	2.4	4.52
24	0	0	2.54	0.74	0.17	3.8	4.30
25	0	0	2.09	0.74	0.5	4.0	4.53
26	0	0	2.08	0.76	0.83	4.1	4.47
27	1	1	2.54	0.60	0.07	2.9	4.79
28	1	1	2.46	0.71	0.07	3.2	4.95
29	2	1	2.59	0.68	0.07	3.3	5.21
30	1	1	1.96	0.71	0.65	3.3	5.11
31	0	1	1.85	0.70	99	2.8	5.19
32	0	1	2.41	0.70	0.10	3.2	4.01
33	0	0	1.85	0.66	0.00	3.4	4.25
34	0	0	1.43	0.69	0.00	3.3	4.04
35	0	0	1.64	0.64	0.01	3.4	4.19
36	0	0	2.19	0.81	0.00	4.5	4.54
37	0	0	2.33	0.77	0.03	4.4	4.81
38	1	0	1.92	0.62	0.12	3.2	4.57
39	0	0	2.24	0.82	0.01	4.3	4.14
40	0	0	2.02	0.76	0.00	3.7	4.77
41	0	0	1.80	0.71	0.41	4.2	3.88

MW: molecular weight (Da); nHBD: number of hydrogen bond donors; TPSA: topological polar surface area (Å²); CLogP: consensus logarithm of the octanol/water partition coefficient; BBB: blood-brain barrier penetration probability; PgP_sub: P-glycoprotein substrate probability; Lipinski #violations: number of violations of Lipinski's rule of five; Ghose #violations and Veber #violations: violations of Ghose and Veber rules; logD: pH-dependent distribution coefficient (pH 7.4); QED: quantitative estimate of drug-likeness; LM-human: fraction remaining after 30 min in human liver microsomes; CNS MPO: Pfizer CNS multiparameter optimization score; SA: synthetic accessibility score (1 = easy, 10 = difficult). The full chemical name of OGH is given in the main text, Section 2.3.

Table S5. Comparative docking and activity data for compounds 5 and 6: structural basis for the effect of difluoro substitution on BACE1 inhibition.

Feature	Compound 5	Compound 6
pIC_{50} (experimental)	8.80	7.82
ΔG (kcal/mol)	–9.0	–8.2
Conventional H-bonds	5	1
Halogen bonds (F)	2 (GLN60, GLY278)	3 (ARG176, TYR246, PRO118)
Pi-cation	Yes (ARG283)	No
Pi-stacking distance (TYR119)	5.86 Å	4.49 Å
Key H-bond partners	THR280, GLN121, LYS155 (×2), PHE156	TYR119 (only)

ΔG: binding free energy (kcal/mol). H-bonds: conventional hydrogen bonds. Distances for π-stacking are given in Ångström (Å). Interaction types (e.g., hydrogen bonds, halogen bonds, π-cation, π-alkyl) are defined in the legend of Figure 4 in the main text (which applies to all interaction maps in this study).

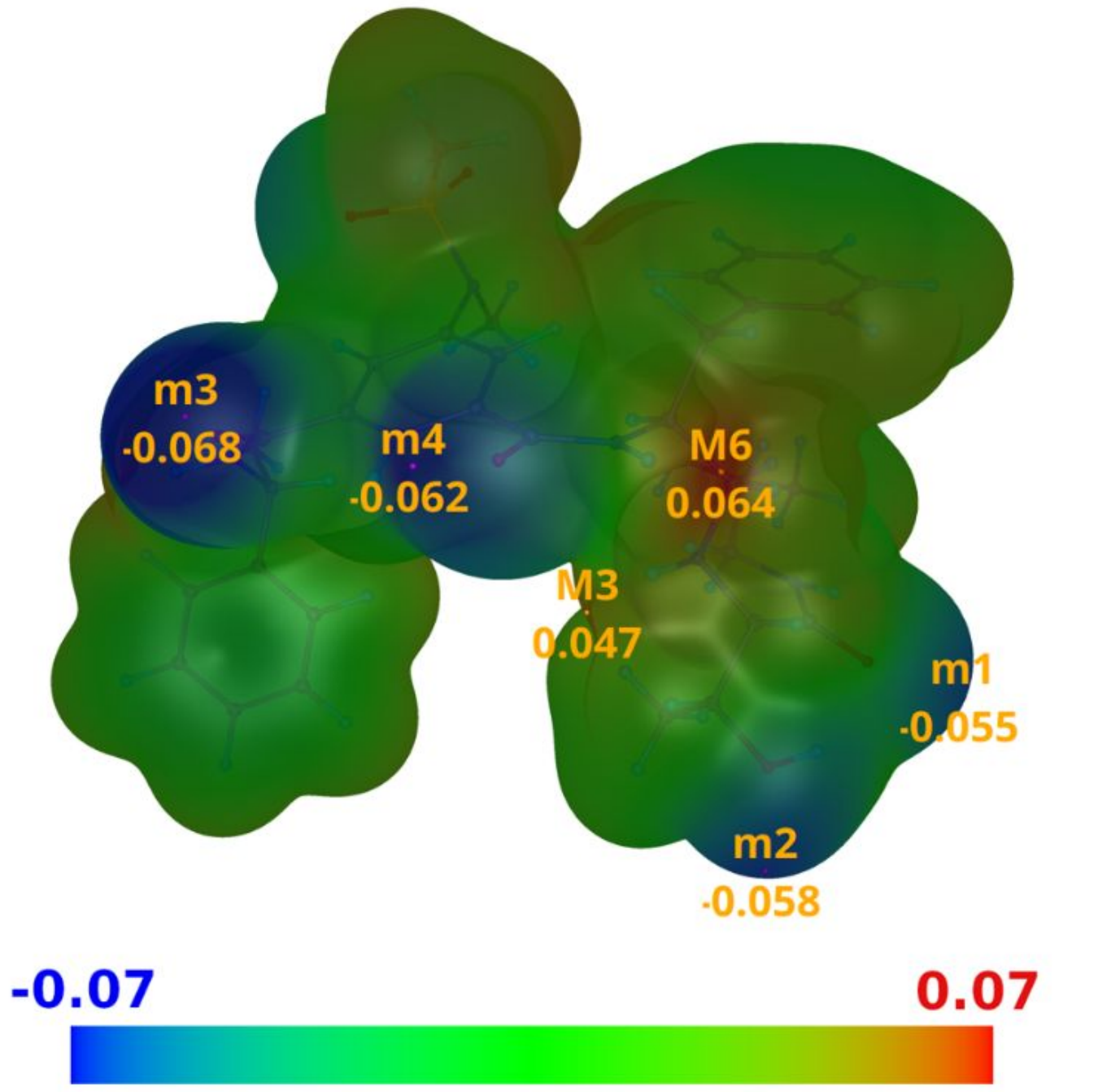

Reference Compound OGH Isolated

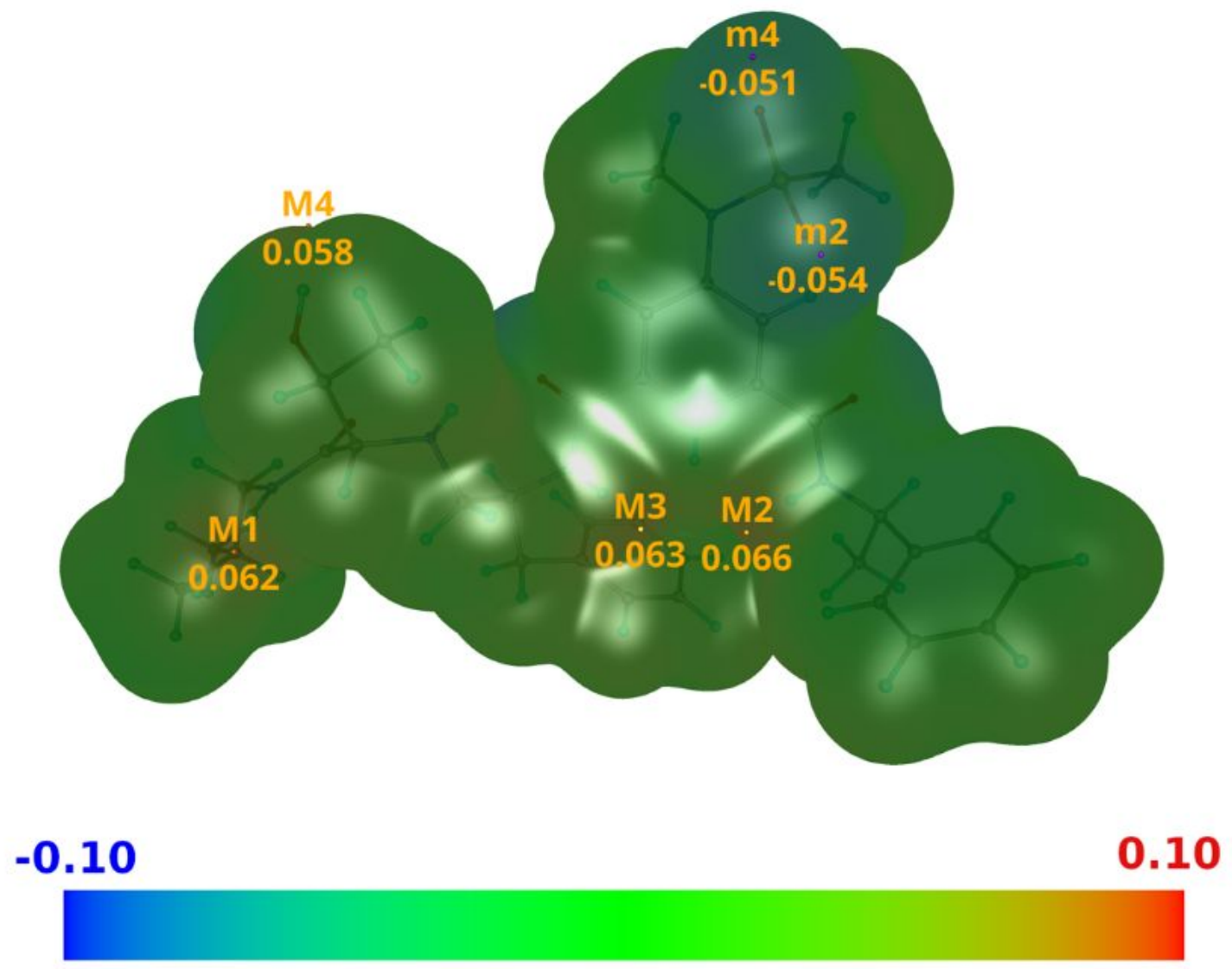

Reference Compound OGH Embedded

Figure S1. Molecular electrostatic potential (MESP) maps of reference compound OGH in its isolated conformation and in the conformation, it adopts when binding to the BACE1 active site. The MESP was computed on the electron density isosurface (0.001 a.u.). Blue regions indicate negative potential (electron-rich), red regions indicate positive potential (electron-deficient), and green indicates neutral. The positions of local MESP maxima (blue dots) and minima (red dots) are shown. Top: isolated (gas-phase optimized) conformation, exhibiting Nmin = 6 and Nmax = 9. Bottom: conformation extracted from the docking pose (embedded), showing Nmin = 4 and Nmax = 4, reflecting geometric constraints imposed by the BACE1 active site.

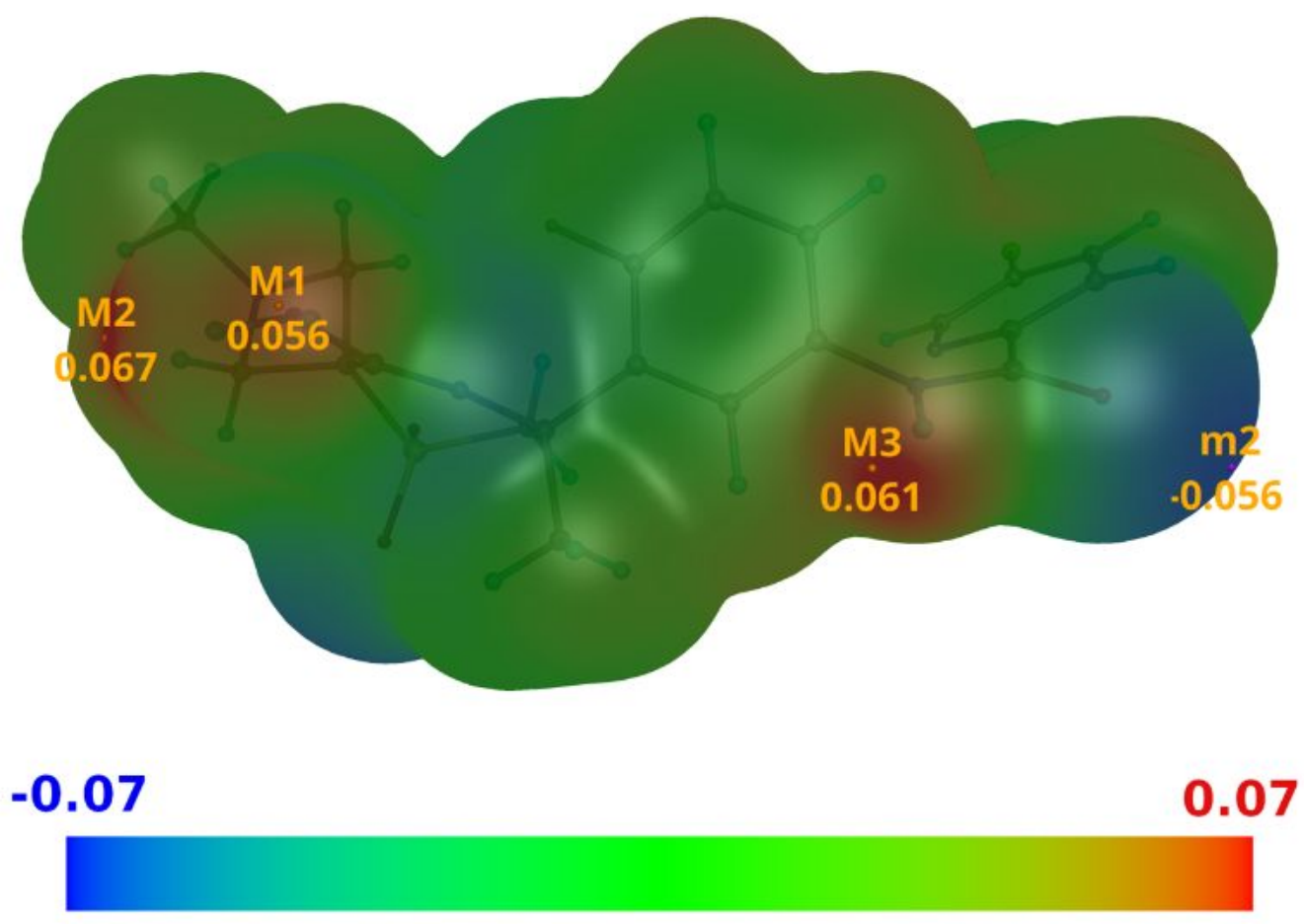

Compound 20 Isolated

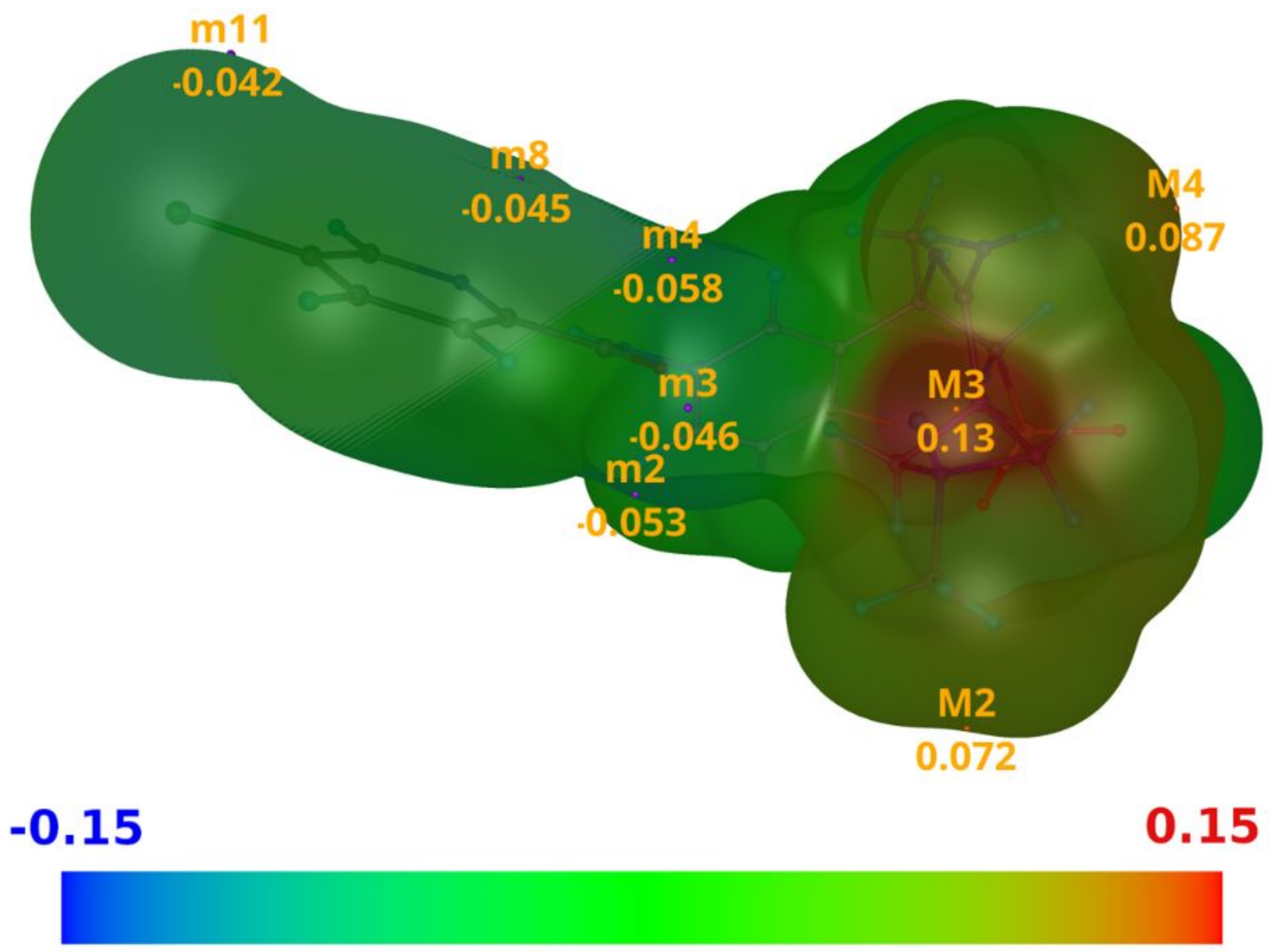

Compound 20 Embedded

Figure S2. Molecular electrostatic potential (MESP) maps of compound 20 in its isolated conformation and in the conformation, it adopts when binding to the BACE1 active site. The MESP was computed on the electron density isosurface (0.001 a.u.). Blue regions indicate negative potential (electron-rich), red regions indicate positive potential (electron-deficient), and green indicates neutral. The positions of local MESP maxima (blue dots) and minima (red dots) are shown. Top: isolated (gas-phase optimized) conformation, exhibiting Nmin = 4 and Nmax = 3. Bottom: conformation extracted from the docking pose (embedded), showing Nmin = 11 and Nmax = 4, reflecting the structural rearrangement induced by the BACE1 active site.
